# Multiyear in-situ L-band microwave radiometry of land surface processes on the Tibetan Plateau

**DOI:** 10.1038/s41597-020-00657-1

**Published:** 2020-09-30

**Authors:** Z. Su, J. Wen, Y. Zeng, H. Zhao, S. Lv, R. van der Velde, D. Zheng, X. Wang, Z. Wang, M. Schwank, Y. Kerr, S. Yueh, A. Colliander, H. Qian, M. Drusch, S. Mecklenburg

**Affiliations:** 1 Faculty of Geo-information Science and Earth Observation (ITC), University of Twente, Enschede, The Netherlands; 2 College of Atmospheric Sciences, Plateau Atmosphere and Environment Key Laboratory of Sichuan Province, Chengdu University of Information Technology, Chengdu, China; 3 Institute of Tibetan Plateau Research, Chinese Academy of Sciences, Beijing, China; 4 Key Laboratory of Land Surface Process and Climate Change in Cold and Arid Regions, Northwest Institute of Eco-Environment and Resources, Chinese Academy of Sciences, Lanzhou, China; 5 Swiss Federal Research Institute WSL, Birmensdorf, Switzerland; 6 Gamma Remote Sensing AG, Gümligen, Switzerland; 7 CESBIO (CNES/CNRS/UPS/IRD), Toulouse, France; 8 Jet Propulsion Laboratory, Pasadena, USA; 9 Key Laboratory of Subsurface Hydrology and Ecological Effect in Arid Region of Ministry of Education, School of Water and Environment, Chang’an University, Xi’an, 710054 China; 10 European Space Agency, ESTEC, Earth Observation Programmes, Noordwijk, The Netherlands; 11 European Space Agency, ESA Climate Office, Harwell Campus, Oxfordshire, UK

**Keywords:** Cryospheric science, Hydrology

## Abstract

We report a unique multiyear L-band microwave radiometry dataset collected at the Maqu site on the eastern Tibetan Plateau and demonstrate its utilities in advancing our understandings of microwave observations of land surface processes. The presented dataset contains measurements of L-band brightness temperature by an ELBARA-III microwave radiometer in horizontal and vertical polarization, profile soil moisture and soil temperature, turbulent heat fluxes, and meteorological data from the beginning of 2016 till August 2019, while the experiment is still continuing. Auxiliary vegetation and soil texture information collected in dedicated campaigns are also reported. This dataset can be used to validate the Soil Moisture and Ocean Salinity (SMOS) and Soil Moisture Active Passive (SMAP) satellite based observations and retrievals, verify radiative transfer model assumptions and validate land surface model and reanalysis outputs, retrieve soil properties, as well as to quantify land-atmosphere exchanges of energy, water and carbon and help to reduce discrepancies and uncertainties in current Earth System Models (ESM) parameterizations. Measurement cases in winter, pre-monsoon, monsoon and post-monsoon periods are presented.

## Abstract (summary)

**Table:** 

Measurement(s)	microwave radiation • soil moisture • atmospheric wind • Solar Radiation • rain • soil • CO2/H2O flux • thermal radiation • temperature of soil
Technology Type(s)	Radiometry (Microwave) • Sensor Device • pyranometer • Gauge or Meter Device • particle size analyzer • pyrgeometer
Sample Characteristic - Environment	land
Sample Characteristic - Location	Tibetan Plateau

Machine-accessible metadata file describing the reported data: 10.6084/m9.figshare.12818261

## Background & Summary

Microwave remote sensing of land surfaces on a global scale has mainly focused on soil moisture retrieval in the recent past. This is because soil moisture strongly influences hydrological and agricultural processes in controlling runoff generation, drought development, and agroecosystem functioning. As a source of water for evaporation and transpiration over land surfaces, soil moisture is involved in the water, energy and carbon cycles of the Earth system and impacts on the climate system through atmospheric feedbacks. Due to its significance, soil moisture was recognized as an Essential Climate Variable (ECV) by the Global Climate Observing System (GCOS) in 2010, and several international programs have been established in recent years to produce global soil moisture data. They include the Climate Change Initiative (CCI) of the European Space Agency (ESA), the Soil Moisture and Ocean Salinity (SMOS^1^) satellite mission of ESA, and the Soil Moisture Active Passive (SMAP^2,3^) satellite mission of the National Aeronautics and Space Administration (NASA). The two dedicated satellite missions for observation of soil moisture SMOS and SMAP provide global soil moisture products at nearly daily temporal resolution and coarse spatial resolution (e.g., 15/25 km for SMOS and 36 km for SMAP).

Despite these advances, the operational retrieval algorithms have relied on zeroth-order radiative transfer theory (the so-called τ-ω model) and empirical assumptions in passive microwave retrievals in the past. Although more sophisticated approaches are currently being developed to account for multiple scattering, in particular over forested areas^4–7^, there is still little consensus about the effects of vegetation interception and litter on observed brightness temperature, which remains difficult to be addressed by studies based on space-borne observations from passive microwave satellite data^8^. As an example, soil moisture retrieval using both SMAP vertical and horizontal polarization does not outperform the use of single vertical polarization^9^. Similarly, the precise nature of the vegetation scattering and emission and its representation in active microwave retrievals have remained unresolved, resulting in the current largely empirical approaches and large uncertainties and inconsistencies among different operational soil moisture products^10,11^. As a further complication, when a land surface undergoes freeze-thaw processes, the behaviour of microwave observation abruptly changes in response to changes in the phase of the soil water (i.e., liquid or solid phase) at different soil depths. While such a dynamic process in space and time can be observed and modelled with *in situ* measurements^12–14^, current satellite retrievals can only provide freeze-thaw information (date and range of depths) using passive microwaves at a low resolution^15,16^ or a binary indication of the frozen or thawed surfaces^17^. All these results point to a fundamental need to advance knowledge in understanding the precise scattering-emission mechanism of vegetated lands and the need for in-depth investigations of freeze-thaw processes.

**Fig. 1:**
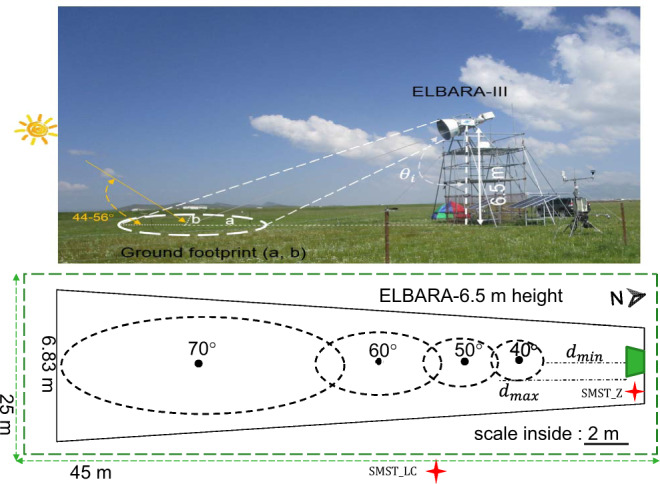
A schematic overview of the ELBARA-III tower setup (top panel) and the footprints (bottom panel). The footprints vary from 3.31 m^2^ to 43.64 m^2^ for incidence angle from 40° to 70°. The half-axes of the elliptic footprint are indicated as *a* and *b* for a given incidence angle ${\theta }_{i}$ (top panel) and the projected ground distances from the radiometer to the closest- and the farthest-side of the elliptic footprints at −3 dB sensitivity of the antenna are indicated as *d*_min_ and *d*_max_ (bottom panel). The locations of the installed *in situ* soil moisture and soil temperature sensors are indicated as SMST_Z and SMST_LC. The fence (25 m × 45 m) is not drawn to scale.

To contribute to filling this knowledge gap, an L-band microwave observation system (ELBARA-III; See Fig. 1) was set up at the Maqu site of the Tibetan soil moisture and soil temperature observation network^10^ for long-term observation of the land surface processes since the beginning of 2016. ELBARA-III is known as the third generation ESA L-Band Radiometer. The collected dataset contains measurements of L-band brightness temperature in horizontal and vertical polarization, profile soil moisture and soil temperature, turbulent heat fluxes, and meteorological data. Auxiliary vegetation and soil texture information were also collected during intensive field campaigns. This dataset can be used to validate SMOS and SMAP brightness temperature observations and retrievals, verify radiative transfer model assumptions and validate land surface model and reanalysis outputs, retrieve soil properties, as well as to quantify land-atmosphere exchanges of energy, water and carbon that are essential in realistic Earth system modeling. Figure 1 provides a schematic overview of the ELBARA-III radiometry setup.

## Methods

The dataset is collected at the Maqu regional-scale soil moisture and soil temperature (SMST) monitoring network on the north-eastern Tibetan Plateau^10,18^ and it mainly contains measurements of L-band brightness temperature in horizontal and vertical polarization by an ELBARA-III microwave radiometer, profile soil moisture and soil temperature by 5TM sensors, turbulent heat fluxes by a CSAT eddy covariance system, and meteorological data by an automatic weather station. Auxiliary vegetation and soil texture information were also collected during intensive field campaigns.

The Maqu SMST monitoring network (33°30′–34°15′ N, 101°38′–102°45′ E) is located in the source region of the Yellow River on the north-eastern part of the Tibetan Plateau at an altitude between 3200 m and 4200 m above mean sea level. The Maqu area has a cold climate with dry winter and warm summer (Dwb) in the updated Köppen-Geiger climate classification^19^. Land cover is mainly alpine meadows with grass heights varying from 5 to 15 cm throughout the growing season due to intensive grazing by livestock. The network is equipped with 20 profile measurements of SMST distributed over an area of 40 km by 80 km. The ELBARA-III radiometer is installed at the center of the SMST monitoring network (hereafter Maqu site) to collect continuous microwave radiometric signatures of the grassland site. The prevailing soil types are sandy loam, silt loam, and organic soil with on average ~30.3% sand, ~9.9% clay, and a maximum of ~39.0% organic matter based on soil sampling^20^.

### L-band brightness temperature

ELBARA-III is an L-band (1.4 GHz) Dicke-type radiometer with a dual-polarized conical horn antenna with −3 dB beam width of 12°. It uses a resistive load (RL), and an active cold load (ACL) as internal calibration sources to derive calibrated brightness temperature (${T}_{b}^{p}$) of the ground footprints in horizontal (*p* = *H*) and vertical polarization (*p* = *V*). The 50 Ω RL is kept at a stabilized instrument internal temperature *T*_inst_ to better than ±0.1 K to ensure the noise temperature *T*_RL_ = *T*_inst_. The ACL is a low-noise amplifier with its noise temperature *T*_ACL_ calibrated by cold sky measurements. To mitigate and detect potential radio frequency interference (RFI), the radiometric signal is split into two sub-bands, one in 1.402–1.413 GHz and the other in 1.414–1.425 GHz, within the protected 1.400–1.427 GHz of the microwave L-band (1–2 GHz). The absolute accuracy of ELBARA-III ${T}_{b}^{p}$ measurement is better than 1 K and the corresponding sensitivity is at least 0.1 K. ELBARA-III has upgraded components and functions compared to ELBARA-II^21^ including a new temperature-controlled Radiometer Microwave Assembly (RMA), a new thermoelectric cooling (TEC) controller for the Peltier elements, a new weather resistant Instrument Computer (IC) enclosure, a new detector assembly, longer antenna feed cables (1 m), addition of a 6 dB attenuator at the input of the sub-band filters to improve isolation between the filters which reduces the leakage from one filter to the other by 12 dB, and a bootloader of the TEC allowing upgrading firmware from the IC.

The ELBARA-III measurement cycle consists of successive measurements of the ambient, hot and cold loads followed by the H and V antenna polarization channels. At each position the total power of the radiometer is measured for a period of approximately 4 seconds. The measurements of the ambient and cold load establish the calibration line for the radiometer. The slope of the line is the radiometer gain and the intercept is related to the noise generated by the radiometer itself. Once the internal noise temperatures are calibrated the brightness temperature ${T}_{b}^{p}$ at polarization *p* = *H,V* can be computed from the measured radiometer voltages. The raw data ${U}_{in}^{p}$ is measured when the input switch is on the antenna *H* or *V* polarization position, which needs to be corrected with the unavoidable losses from the feed cable. The noise temperature at the radiometer input can be computed as ${T}_{b\,in}^{p}$ = (*T*_*RL*_ - *T*_*ACL*_) (${U}_{in}^{p}$ - *U*_*ACL*_)/(*U*_*RL*_ - *U*_*ACL*_ ) + *T*_*ACL*_, where *U*_*RL*_, *U*_*ACL*_ are the voltages measured when the input switch is set to the resistive load and the active cold load, and *T*_*RL*_ and *T*_*ACL*_ are noise temperatures related to calibration sources and are internally calculated. After considering the contribution of the feed cable loss, the brightness temperature is calculated from the raw data as ${T}_{b}^{p}=[{T}_{b\,in}^{p}-(1-{\tau }_{fc}){T}_{fc}]/{\tau }_{fc}$, where ${\tau }_{fc}$ is the transmissivity of the feed cable and ${T}_{fc}$ equals to the ambient temperature of the feed cable.

The ELBARA-III radiometer is installed at Maqu site on a 4.8 m high scaffold tower, making the center of rotation at 6.5 m in height above ground with the antenna pointing towards the south. The continuous measurements include angular scanning of the ground at every 30 min and sky measurements. The angular range of the ground scans is between 40°−70° (relative to nadir) in steps of 5°, and sky measurements are performed at 23:55 local time every day with an observation angle of 155° (relative to nadir). The elliptic footprints at −3 dB sensitivity of the antenna are estimated according to the installation height and the incidence angle^22^ (Fig. 1).

### Profile soil moisture and soil temperature

SMST profiles are automatically measured by 5TM ECH_2_O probes (METER Group, Inc. USA) next to the radiometer tower at 15-min time-intervals installed at the depths for SMST-Z profile^13^: 5, 10, 20, 40, 80 and 160 cm (one sensor per depth) and for SMST-LC profile^23^: 2.5 cm (2 sensors), then one sensor every 2.5 cm in the top 20 cm, every 5 cm between 20–50 cm and every 10 cm between 50–100 cm (19 layers and 20 sensors in total). The 5TM probe is a capacitance sensor operating at 75 MHz measuring the dielectric permittivity of the surrounding soil, and the measured dielectric permittivity values are converted to volumetric liquid water contents using the Topp equation^24^. Specific calibrations were conducted for the profile soil textures^18,20^.

### Turbulent heat fluxes

The micro-meteorological observations are conducted by an eddy-covariance (EC, CSAT3) system which is installed for measuring the turbulent heat fluxes.

### Meteorological data

The meteorological observation system consists of an automatic weather station which provides wind speed and direction, air humidity and temperature measurements at 2.5 m above ground, and instrumentations for measuring four radiation components (i.e., up- and downwelling shortwave and longwave radiations), as well as air pressure and liquid precipitation (Geonor T-200B Series). The albedo is calculated as the ratio of up- and downwelling shortwave radiations, and the surface temperature is derived from the up- and downwelling longwave radiations.

### Soil and vegetation data

Soil samples are collected around the ELBARA-III field site for laboratory analyses to quantify the soil hydraulic and thermal properties, i.e., soil texture (sand, clay, and silt), organic matter content, bulk density, porosity, soil water potential at air-entry and empirical parameters related to the pore-size distribution of the soil matrix. Field measurements of the saturated hydraulic conductivity are also carried out. The detailed analysis and measured hydraulic properties are given in^13,20^.

The MODIS leaf area index (LAI) product derived from data by the Terra and Aqua satellites (MCD15A2H^25^) is extracted to represent the vegetation status. The time series of LAI is processed with the harmonic analysis of the time series (HANTS) algorithm^26^ to remove cloud contaminations. Additional field measurements of fresh and dry above-ground biomass, LAI, and vegetation height were conducted during a field campaign in 2018^27^.

## Data Records

We present here the collected Maqu ELBARA-III radiometry dataset^28,29^ in detail including L-band brightness temperature, profile soil moisture and soil temperature, turbulent heat fluxes, meteorological data, as well as soil and vegetation data. Online-only Table 1 summarizes the general characteristics of the Maqu site, the included variables in the data, the used instruments and their setups. Table 1 presents the availability of the collected data by different instrumentations.

**Table 1:** Overview of data availability.

Data Type	2016–2017 period					
	StartT	EndT	StartT	EndT	StartT	EndT
Meteo data^*^	25-Mar-2016					30-Jan-2017
Eddy covariance			5-Jun-2016	4-Sep-2016	1-Dec-2016	29-Mar-2017
SMST_LC			7-Aug-2016			29-Mar-2017
SMST_Z	1-Jan-2016	6-Apr-2016				
ELBARA TB	1-Jan-2016	6-Apr-2016	7-Aug-2016	30-Nov-2016	1-Jan-2017	29-Mar-2017
*In-situ* LAI	—					
MODIS LAI	From Jan-2016 to Dec-2019					
SMAP L1 TB	From 1-Jan-2016 to 31-Dec-2019					
**Data Type**	**2017–2018 period**					
	**StartT**	**EndT**	**StartT**	**EndT**		
Meteo data	31-Jul-2017	27-Aug-2017	22-Oct-2017	12-Aug-2018		
Eddy covariance	29-Mar-2017	16-Nov-2017	9-Dec-2017	12-Aug-2018		
SMST_LC	27-Jul-2017			12-Aug-2018		
SMST_Z	—					
ELBARA TB	29-Mar-2017			12-Aug-2018		
*In-situ* LAI	12-Jul-2018, 17-Aug-2018					
MODIS LAI	From Jan-2016 to Dec-2019					
SMAP L1 TB	From 1-Jan-2016 to 31-Dec-2019					
**Data Type**	**2018–2019 period**					
	**StartT**	**EndT**	**StartT**	**EndT**		
Meteo data	12-Aug-2018	30-Oct-2018	11-Nov-2018	28-Aug-2019		
Eddy covariance	12-Aug-2018	10-Nov-2018	26-Mar-2019	28-Aug-2019		
SMST_LC	15-Aug-2018			31-May-2019		
SMST_Z	—					
ELBARA TB	12-Aug-2018	29-Dec-2018	25-Mar-2019	28-Aug-2019		
*In-situ* LAI	12-Jul-2018, 17-Aug-2018					
MODIS LAI	From Jan-2016 to Dec-2019					
SMAP L1 TB	From 1-Jan-2016 to 31-Dec-2019					

(StartT: start of a data period, EndT: end of a data period; Meteo data: meteorological data; Eddy covariance: micrometeorological data; SMST_LC: Soil moisture and soil temperature data at LC location^23^, SMST_Z: Soil moisture and soil temperature at Z location^13^; ELBARA TB: ELBARA-III brightness temperature; In-situ LAI: in-situ measured leaf area index^27^; MODIS LAI: Leaf area index from the MODIS sensor^25^; SMAP L1 TB: SMAP Level 1 brightness temperature^3^).

(*The precipitation data from 1-Sep-2016 to 30-Jan-2017 was provided by the Zoige Plateau Wetlands Ecosystem Research Station, Northwest Institute of Eco-Environment and Resources, Chinese Academy of Science, Lanzhou, China; The precipitation gauge is the same type Geonor T-200B Series installed 200 m to the north-west of Maqu site).

The dataset as described in Table 1 can be accessed at https://figshare.com. Detailed technical description of the data records can be found in the readme.txt files and codes and procedures for processing and plotting the figures, and for downloading satellite data are also included. The contents of the two figshare data records^28,29^ are described in Online-only Table 2 and Table 2, respectively.

**Table 2:** Codes for filtering brightness temperature (TB) outliers and TB data with corrected local time^29^.

Directory	Subdirectory/File name	Content
Code_update		Code for filtering TB outlier
	TB_FilteringByQuantile.py	Code for quantile filtering
	TB_FilteringByHANTS.py	Code for filtering TB outlier by using HANTS
	Plot_DailyScale_20170701.py	Code for plotting Fig. 2
	AWS_TB_SMST_Display_updated.py	Code for plotting Fig. 4
Data_update		TB data with corrected local time and explanations
	**Data_version1.1**	
	ELBARA-III dataset-2018–2019ELBARA-III TB.csv	ELBARA TB data, 2018–2019
	ELBARA-III dataset-2017–2018ELBARA-III TB.csv	ELBARA TB data, 2017–2018
	ELBARA-III dataset-2016–2017ELBARA-III TB.csv	ELBARA TB data, 2016–2017
	SMAP_ATBD_TimeInfor.pdf	SMAP Time information
	README_Version1.1.txt	README file for version 1.1
	Note on Filtering brightness temperature caused by solar reflection_v1.2.docx	Note on filtering TB data
	Maqu_NoonTime.dat	Local noon time at Maqu

## Technical Validation

We present here analyses to support the technical quality of the Maqu microwave radiometry dataset. While the used instrumentation and the data collection have been presented in the methods section, this section focuses on the consistency of the different variables that enable further exploration and application of the data.

The quality assurance and quality control (QA/QC) of ELBARA-III measurements are carried out through the ‘histogram test’ on the voltage samples (raw-data) of the detector output at sampling frequency of 800 Hz. The ‘histogram test’ is an end-to-end test of the radiometer. Specifically, statistics of the histogram test can show if there is an internal non-Gaussian Radio Frequency Interference (RFI) via excess kurtosis and standard deviation. Furthermore, serious imbalances between the two receiver channels (for each polarization) can be identified via mean values. Another QA/QC test is via the ‘sky-looking’ measurement. If the voltages at the antenna ports measured during sky measurements are close to each other, it indicates that the losses in the two antenna cables (i.e., H and V polarization) are almost the same (meaning stable ELBARA-III operations). Other indicators used for QA/QC purposes include the instrument internal temperature, active cold source temperature, ambient temperature, and the angular behaviour of the processed brightness temperatures. These QA/QC procedures are carried out routinely after data downloading and have indicated excellent performance of the ELBARA-III radiometer. There is no potential (non-Gaussian) RFI detected at the Maqu site during the ELBARA-III operation and the ELBARA-III measurements appear reliable and reflect the environmental conditions of the observed footprint areas.

**Fig. 2:**
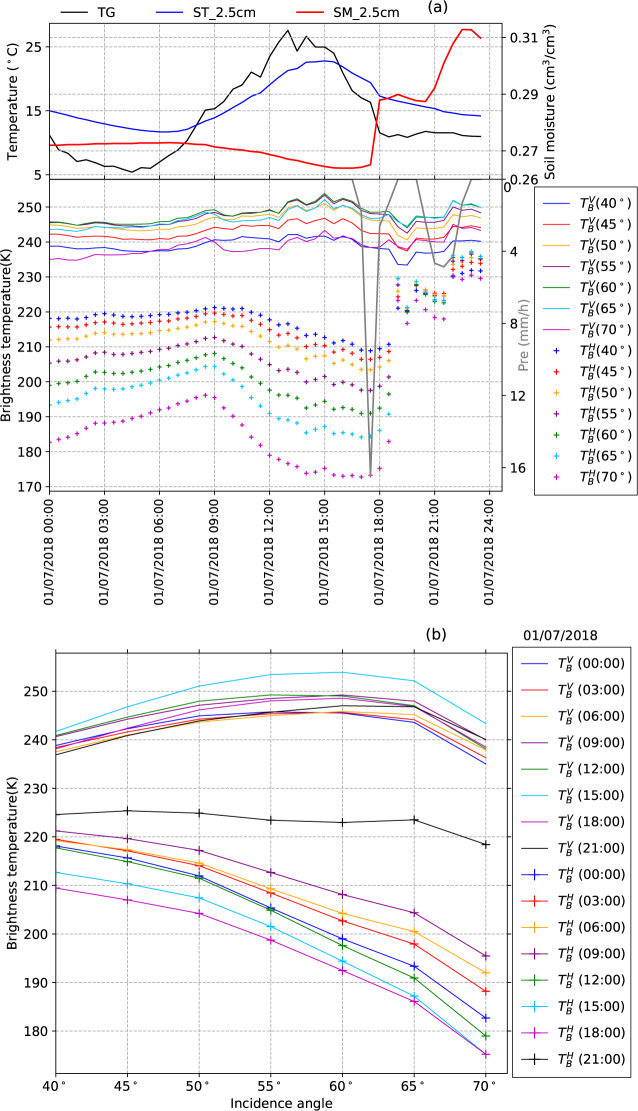
Angular variations of the Maqu ELBARA-III radiometry dataset for 01/07/2018 (date is given in dd/mm/yyyy). (**a**) plotted are ground surface temperature (TG), soil temperature at 2.5 cm depth (ST_2.5 cm), and soil moisture at 2.5 cm depth (SM_2.5 cm) (top panel), and the brightness temperature in horizontal and vertical polarization (${T}_{b}^{H}$, ${T}_{b}^{V}$) from 40° to 70° incidence angle and precipitation (Pre) (bottom panel). (**b**) angular plot of (${T}_{b}^{H}$, ${T}_{b}^{V}$) at 3 hours intervals.

Figure 2 shows an example of angular variations of the Maqu ELBARA-III radiometry dataset for 01/07/2018 (date is given in dd/mm/yyyy). The presented variables in Fig. 2(a) are ground surface temperature (TG), soil temperature at 2.5 cm depth (ST_2.5 cm), and soil moisture at 2.5 cm depth (SM_2.5 cm) (top panel) to assist the interpretation of the observed brightness temperature in horizontal and vertical polarization (${T}_{b}^{H}$, ${T}_{b}^{V}$) from 40° to 70° incidence angle in combination with precipitation (Pre) (bottom panel). TG and ST_2.5 cm present a sinusoidal variation with ST_2.5 cm lagging TG by about one hour and having a smaller amplitude. SM_2.5 cm shows a gradual decrease from early morning (6:00) until the heavy precipitation just before 18:00 after which it jumps from ca. 0.26 to 0.29 m^3^/m^3^, and then to ca. 0.31 m^3^/m^3^ after a second precipitation event around 21:00. ${T}_{b}^{H}$ and ${T}_{b}^{V}$ present typical angular variations (Fig. 2(b)) with ${T}_{b}^{H}$ decreasing from 9:00 till 18:00 and strongly reacting to the two precipitation events, while the changes in ${T}_{b}^{V}$ are in general much smaller throughout the whole period. Despite the fact that the precise contributions to ${T}_{b}^{H}$and ${T}_{b}^{V}$from soil and vegetation emission and their interaction need to be quantified by more detailed modeling, we select a few characteristic periods from different years to illustrate the panoply of land-atmosphere conditions affecting the ELBARA-III observations at the Maqu site and provide explanations in detail in the following as examples.

**Fig. 3:**
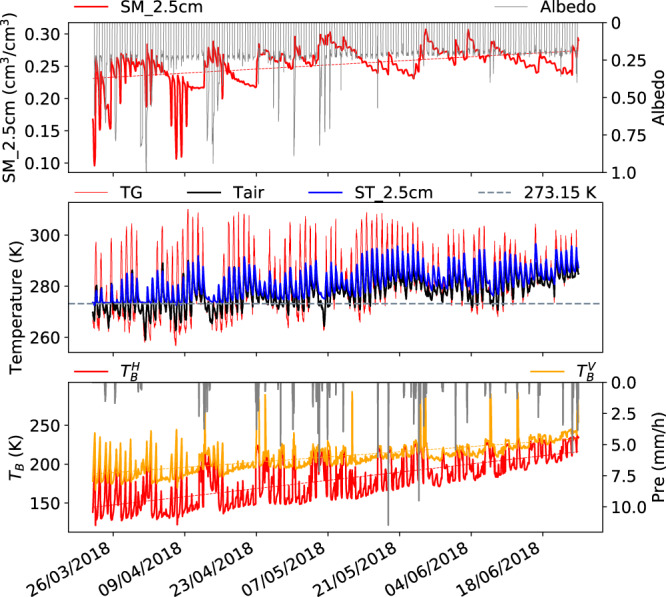
Seasonal variations of the Maqu ELBARA-III radiometry dataset for pre-monsoon season (late March to late June) in 2018. Plotted are soil moisture at 2.5 cm depth (SM_2.5 cm), albedo, ground surface temperature (TG), air temperature (Tair), soil temperature at 2.5 cm depth (ST_2.5 cm), the nominal freezing point as a reference (273.15 K), and the brightness temperature in horizontal and vertical polarization (${T}_{b}^{H}$, ${T}_{b}^{V}$) at 40° incidence angle and precipitation (Pre). Trend lines (dashed lines) are added to SM_2.5 cm and (${T}_{b}^{H}$, ${T}_{b}^{V}$) time series to assist interpretation.

**Fig. 4:**
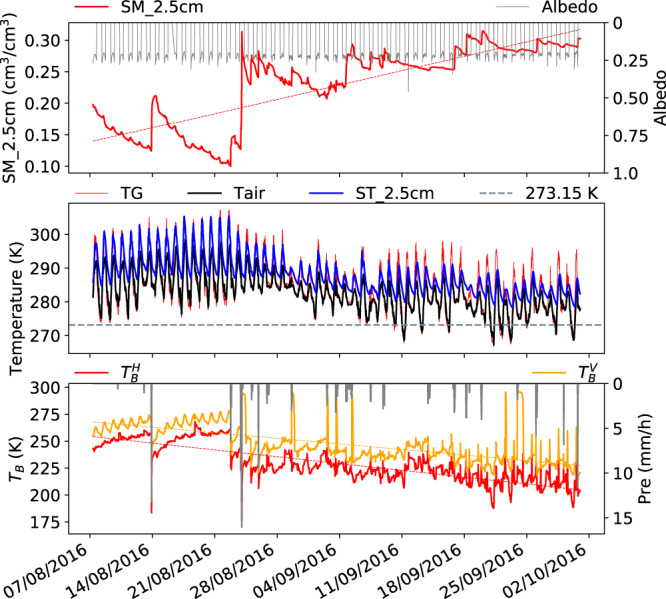
Same as Fig. 3 but for monsoon period (August to late September) in 2016.

**Fig. 5:**
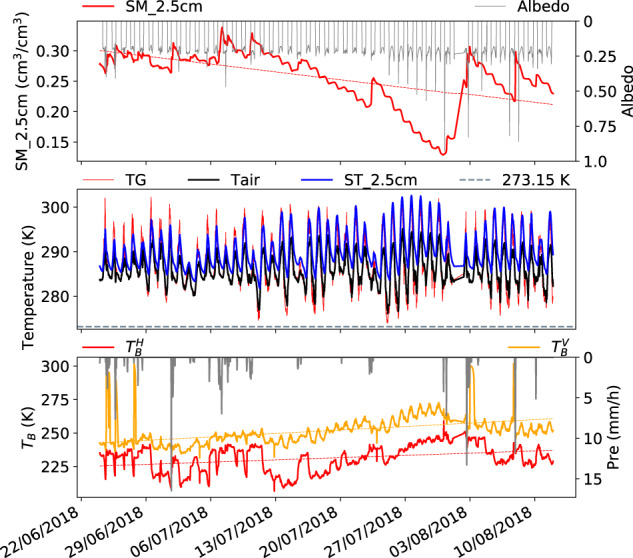
Same as Fig. 3 but for monsoon period (late June to mid-August) in 2018.

**Fig. 6:**
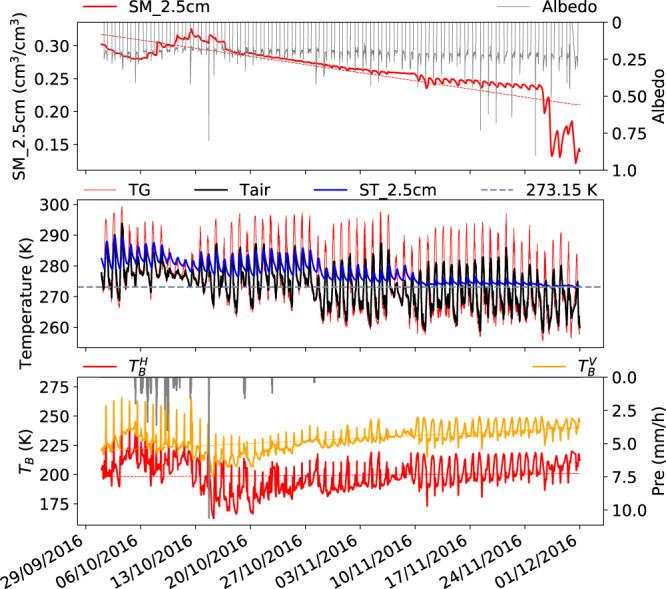
Same as Fig. 3 but for post-monsoon (early October to late November) in 2016.

**Fig. 7:**
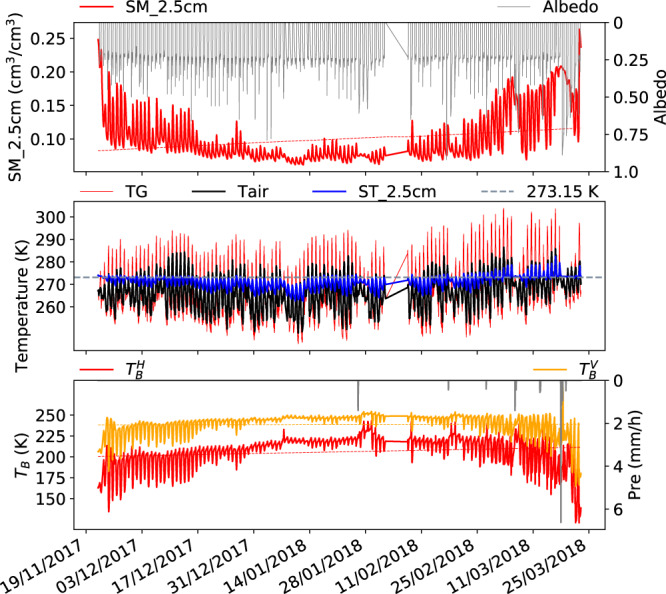
Same as Fig. 3 but for winter season (late November to late March) in 2017–2018.

**Fig. 8:**
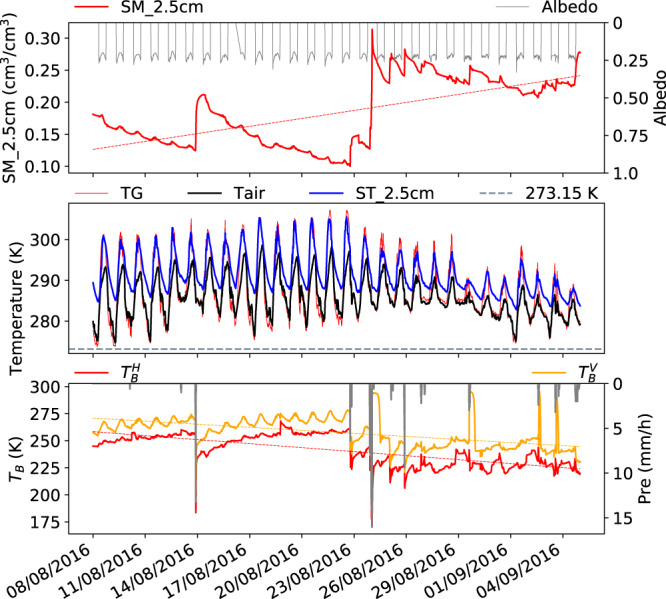
Diurnal dynamics of the Maqu ELBARA-III radiometry dataset for 08/08/2016–04/09/2016 monsoon period. Plotted variables are the same as in Fig. 3, except diurnal characteristics in different seasons are highlighted.

Figures 3–6 present samples of seasonal variations of the Maqu ELBARA-III microwave radiometry dataset for different seasons, including pre-monsoon (late March to late June) (Fig. 3), monsoon (late June to late September) (Figs. 4–6), post-monsoon (early October to late November) (Fig. 7), and winter season (late November to late March) (Fig. 8). For each figure, three panels are plotted to display the ELBARA-III brightness temperature observations in horizontal and vertical polarization at 40° incidence angle, together with precipitation in panel 3 and other most relevant variables that can be used to explain the observed variations in the brightness temperature in panels 1 and 2. Panel 1 displays soil moisture at 2.5 cm depth and albedo, and panel 2 displays ground surface temperature (TG), air temperature (Tair), soil temperature at 2.5 cm depth (ST_2.5 cm), and the nominal freezing point as a reference (273.15 K). Both panels support to appreciate and understand the variations in the ELBARA-III observations in terms of environmental variables.

In the pre-monsoon period in 2018, the gradual increase of (${T}_{b}^{H}$, ${T}_{b}^{V}$) (panel 3, the bottom panel in Fig. 3) is due to the increasing of soil temperature (panel 2, resulting in increasing effective temperature^30^) and increase of vegetation which is indicated by the decreasing difference between ${T}_{b}^{H}$ and ${T}_{b}^{V}$. The soil moisture at 2.5 cm depth (SM_2.5 cm) changed gradually from approximately 0.23 m^3^/m^3^ in late March to approximately 0.27 m^3^/m^3^ (panel 1), due to thawing of the frozen ground in late March and increasing in precipitation after mid-April. Despite the frequent precipitation events from April to June, the strong evaporation prevented further wetting of the soil profile. The strong evaporation is caused by an increase in solar radiation and a slight reduction of albedo from 0.24 to 0.23 due to the greening of the surface by vegetation. ${T}_{b}^{H}$, ${T}_{b}^{V}$ increased gradually from 140 K to 210 K and from 180 K to 240 K respectively (along the trend line), with some very high values (spikes) in ${T}_{b}^{H}$ and ${T}_{b}^{V}$ the reasons for which will be further explained in the following.

Monsoon period 2016 (Fig. 4): This late monsoon period sees a gradual decrease of (${T}_{b}^{H}$, ${T}_{b}^{V}$) from 250 and 260 K to 210 and 225 K respectively (panel 3) mainly due to the increasing of soil moisture from 0.1 on 23/08/2016 to 0.30 m^3^/m^3^ in September and October (panel 1). The role of vegetation is reduced due to senescence as indicated by the increasing difference between ${T}_{b}^{H}$ and ${T}_{b}^{V}$.

Monsoon period 2018 (Fig. 5): This early monsoon period sees a gradual increase of (${T}_{b}^{H}$, ${T}_{b}^{V}$) from 225 and 240 K to 240 and 260 K respectively (panel 3) with some higher values at the end of July. Because the level of soil moisture remained at around 0.30 m^3^/m^3^ in June, decreasing gradually in July to below 0.15 m^3^/m^3^ and increased to 0.30 after precipitation in August, the increase in (${T}_{b}^{H}$, ${T}_{b}^{V}$) may be attributed to the increase of vegetation biomass. This period is characterized by the strong drying down between precipitation events.

Post-monsoon period 2016 (Fig. 6): The precipitation events in the first half of October caused a reduction in (${T}_{b}^{H}$, ${T}_{b}^{V}$) correspondingly but both gradually increased afterwards from 160 and 200 K (lower bound) to 200 and 230 K. This period is characterized by a gradual decrease of soil moisture from around 0.30 to 0.24 m^3^/m^3^ from 01/10/2016 to 25/11/2016 due to drying and followed by a sharp reduction to 0.11 m^3^/m^3^ on 26/11/2016 due to soil freezing. TG, Tair, and ST_2.5 cm all show a gradual decrease in this period, when ST_2.5 cm touched the freezing point on 26/11/2016 indicating the soil layer from surface to the depth of 2.5 cm is frozen.

Winter period 2017–2018 (Fig. 7): (${T}_{b}^{H}$, ${T}_{b}^{V}$) increased till the end of January and decreased again till the end of March 2018 with strong oscillations at the beginning and end of this period. This period is characterized by a gradual decrease in SM_2.5 cm in late November, a very low SM_2.5 cm of around 0.07 m^3^/m^3^ in the middle of the period due to frozen soil, and a gradual increase of SM_2.5 cm from late February to March. TG and Tair oscillate around the freezing point in the whole period, but ST_2.5 cm gradually moves from above the freezing point at the beginning of this period to below it and later again above it depicting the freezing processes in the winter period.

Next we present in detail some fascicles of diurnal dynamics of the Maqu ELBARA-III microwave radiometry dataset for different periods. The same variables are plotted as in Figs. 2–7, but for a few typical days in different periods with diurnal characteristics in different seasons highlighted. Similarly characteristic features in each sub-figure are explained in detail.

08/08/2016–04/09/2016, monsoon period (Fig. 8): The gradual increase of (${T}_{b}^{H}$, ${T}_{b}^{V}$) after a precipitation event on 14/8/2016 is due to the drying of the soil profile (panel 1) as a consequence of evaporation when the ground temperature (TG), the air temperature (Tair) and soil temperature (ST_2.5 cm) are all above the freezing level (273.15 K), while the sudden drops in (${T}_{b}^{H}$, ${T}_{b}^{V}$) are caused by a decrease of surface emissivity due to the increase of surface soil moisture by precipitation. The spikes in ${T}_{b}^{V}$ (on 24/08/2016, 30/08/2016, and 03/09/2016 and 04/09/2016) are consequences of surface reflected solar beams into the ELBARA-III antenna horn. The fact that such phenomena occur after precipitation events indicates the presence of water on vegetation and soil surfaces. The geometry for such occurrence requires that the solar elevation is in the range of 44–56° (see Fig. 1 for an illustration of such geometry). More information is provided in next section to guide users wishing to filter out these signals.

11/10/2016–29/10/2016, post-monsoon period (Supplementary Figure S1): The occurrence and amount of precipitation decreased in this period (panel 3). After the two precipitation events on 12/10/2016 and 15/10/2016 which caused a gradual decrease of (${T}_{b}^{H}$, ${T}_{b}^{V}$), the soil profile continued the drying (panel 1) as a consequence of evaporation when the ground temperature (TG), the air temperature (Tair) were mostly above the freezing level (273.15 K) during the day but TG and Tair decreased to or below the freezing level at night, while ST_2.5 cm was still above the freezing point both during day and at night. The spikes in ${T}_{b}^{V}$ are still visible but with much reduced amplitude which are mostly below 25 K except on days from 11/10/2016 to 14/10/2016. The much smaller spikes on other days are consequences of melting of surface frozen soil in daytime which is not detected by SM_2.5 cm but can be inferred by the diurnal oscillating TG and Tair around the freezing point in panel 2.

08/08/2017–26/08/2017, monsoon period (Supplementary Figure S2): This monsoon period is similar to that presented in Fig. 8 but showing a delayed first major precipitation event on 20/08/2017 following a prolonged drying down of the soil profile (panel 1).

24/10/2017–14/11/2017, post-monsoon period (Supplementary Figure S3): The period is similar to that in Supplementary Figure S1 but showing a snow event on 29/10/2017–31/10/2017 indicated by the high albedo (0.95 on 29/10/2017 and 0.4 on 31/10/2017). The amplitude of diurnal variations of TG, Tair on these days, and those on 1/11/2017 were much reduced due to the snow event compared to the preceding and the succeeding days. The snow event was a warm snowfall as TG and Tair stayed mostly above the freezing point, and the snow started to melt as seen by the reduced albedo and increased SM_2.5 cm on 29/10/2017 and completely melted on 1/11/2017. The measured mid-day ${T}_{b}^{H}$ jumped by 30 K from 190 K on 28/10/2017 to 220 K on 29/10/2017, while less than 10 K was seen to increase in ${T}_{b}^{V}$ indicating the much bigger sensitivity of ${T}_{b}^{H}$ than ${T}_{b}^{V}$ to the presence of snow on the ground. Other notable features are the freezing events at nights on 13/11/2017 and 14/11/2017 indicated by the reduced SM_2.5 cm (from 0.27 to 0.18 cm^3^/cm^3^ on 13/11/2017 and from 0.26 to 0.17 cm^3^/cm^3^ on 14/11/2017) and the near freezing point ST_2.5 cm (meaning the soil column above it must be partially frozen as TG and Tair were both below the freezing point). The corresponding ${T}_{b}^{H}$ differ by 30 K and ${T}_{b}^{V}$ by 10 K between mid-day and mid-night. It is important to notice that while both ${T}_{b}^{H}$ and ${T}_{b}^{V}$ increased in the snow and freezing events, the causes of the increases are very different. In the former case, the snow contributed to ${T}_{b}^{H}$ by emission, while in the latter one, it was because of the reduction in soil moisture due to freezing that caused the increase of the emissivity. Similar freezing events can also be seen at the nights on 7/11/2017 and 8/11/2017 but with smaller amplitudes.

14/11/2017–08/12/2017, post-monsoon to winter period (Supplementary Figure S4): The freezing-thawing processes continued from 14/11/2017 till 23/11/2017 when the soil layer above 2.5 cm appeared frozen resulting in reduction of measured SM_2.5 cm from 0.25 at mid-day 21/11/2017 to 0.10 cm^3^/cm^3^ at night of 23/11/2017, while it still increased to ca. 0.17 cm^3^/cm^3^ because TG (and on some days Tair) increased to above the freezing point during the mid-day due to the strong solar radiation on the Tibetan plateau. ${T}_{b}^{H}\,{\rm{and}}\,{T}_{b}^{V}$ showed regular patterns modulated by the changes in liquid soil water content governed by freezing-thawing processes (Note TG was always above the freezing point during mid-day).

07/12/2017–31/12/2017, winter period (Supplementary Figure S5): The period is similar to that in Supplementary Figure S4, but for the early winter period. One notable feature is the continuation of the freezing-thawing processes but with reduced amplitude in SM_2.5 cm and the correspondingly reduced amplitude in the variations of ${T}_{b}^{H}\,{\rm{and}}\,{T}_{b}^{V}$ while their levels keep increasing the whole periods.

04/03/2018–01/04/2018, winter to pre-monsoon period (Supplementary Figure S6): In this period, SM_2.5 cm, TG, Tair, and ST_2.5 cm all started to increase. Except during the few snow events, TG and Tair both reached above freezing point during mid-day. The few brief snow events prevented heat transfer to the ground causing TG and as a result Tair to stay below the freezing point for several days (e.g., on 06–07/03/2018, 19/03/2018, 01/04/2018). The melting of the snow and soil ice after 24/03/2018 by increased solar radiation caused SM_2.5 cm to increase to 0.25 cm^3^/cm^3^ during day time. ${T}_{b}^{H}\,{\rm{and}}\,{T}_{b}^{V}$ briefly increased after the snowfall but restored to regular patterns until about 22/03/2018 when the amplitude of variations increased due to the freezing and thawing processes along with the melting of the snow cover and increase of SM_2.5 cm. This variation was only temporarily interrupted by the six small snow events. The biggest diurnal changes in ${T}_{b}^{H}\,{\rm{and}}\,{T}_{b}^{V}$ were 100 K and 50 K respectively on 21–22/03/2018.

02/04/2018–30/04/2018, pre-monsoon period (Supplementary Figure S7): In this period, SM_2.5 cm, TG, Tair, and ST_2.5 cm all increased to above the freezing point most of the time. Noteworthy is the snow event on 13/04/2018, which temporarily reduced TG and Tair but ST_2.5 cm to below the freezing point. The precipitation events in this period were likely wet snowfall that melted during the day, as indicated by the sharp reduction of the albedo during day time. ${T}_{b}^{H}\,{\rm{and}}\,{T}_{b}^{V}$ remained respectively at ca. 150 and 200 K on average in this period except after the snow events when both temporarily increased. The strong variations in both ${T}_{b}^{H}\,{\rm{and}}\,{T}_{b}^{V}$ in 06–09/04/2018 were due to the freezing of the soil at nights as indicated by the variations in SM_2.5 cm. The similar variations in both ${T}_{b}^{H}\,{\rm{and}}\,{T}_{b}^{V}$ in 02–04/04/2018 are due to the melting of the snow indicated by reduction of albedo from 1.0 on 01/04/2018 to 0.5 on 02/04/2018 (panel 1) and refreezing at nights and thawing of the soil during daytime as indicated by SM_2.5 cm and TG, Tair and ST_2.5 cm.

06/07/2018–27/07/2018, monsoon period (Supplementary Figure S8): in this period, TG, Tair and ST_2.5 cm were all about 15 K above the freezing point. The more frequent precipitation events were all in the form of rainfall. ${T}_{b}^{H}\,{\rm{and}}\,{T}_{b}^{V}$ increased respectively to ca. 220 and 240 K on average from 02–12/07/2018. Given that SM_2.5 cm remained at 0.25–0.30 cm^3^/cm^3^ and was similar to the last week of April (Supplementary Figure S7), the increase in both ${T}_{b}^{H}\,{\rm{and}}\,{T}_{b}^{V}$ of 40 and 20 K respectively can only be attributed to vegetation emission because we can assume safely that the surface roughness remained the same from April to July. The increase in ${T}_{b}^{H}\,{\rm{and}}\,{T}_{b}^{V}$ from 215 and 240 K on 13/07/2018 to 245 and 265 K on 30/07/2018 is caused in part by the drying down in this period, but vegetation emission must have also contributed to the increase because of the favorable condition for vegetation growth in the monsoon period (we do notice the reduction of SM_2.5 cm from 0.30 to 0.15 cm^3^/cm^3^ but expect the vegetation have much deeper roots to tap water from deeper soil layers to sustain growth which is also confirmed by the measured leaf area index (LAI) in the fenced area which increased from 3.95 on 12/07/2018 to 7.37 cm^2^/cm^2^ on 17/08/2018.

28/07/2018–11/08/2018, monsoon period (Supplementary Figure S9): The heavier precipitation events of 5–10 mm per hour in this period caused SM_2.5 cm to increase from 0.15 to 0.30 cm^3^/cm^3^ from 28/07/2018 to 03/08/2018. However ${T}_{b}^{H}\,{\rm{and}}\,{T}_{b}^{V}$ remained respectively at 240 and 265 K indicating the dominant role played by vegetation attenuation and emission. After 03/08/2018, ${T}_{b}^{H}\,{\rm{and}}\,{T}_{b}^{V}$ decreased slightly by ca. 10 and 5 K respectively which can be attributed to contributions by the increased SM_2.5 cm to 0.25 cm^3^/cm^3^ on average and a further increase in biomass as indicated above by increased LAI.

## Usage Notes

This dataset can be used to validate satellite based observations and retrievals^31,32^, verify radiative transfer model assumptions^11^ and validate land surface model and reanalysis outputs^14,30^, retrieve soil physical properties, as well as to quantify land-atmosphere exchanges of energy, water and carbon and help to reduce discrepancies and uncertainties in current Earth System Models (ESM) parameterizations.

As reported above, there appear some spikes in the observed ELBARA-III brightness temperature (e.g. on 24/08/2016, 30/08/2016, and 03/09/2016 and 04/09/2016, Fig. 8), which are consequences of surface reflected solar beams into the ELBARA-III antenna horn under certain surface conditions. For users who wish to filter out these signals, we suggest two methods which are briefly described as follows: 1) Quantile filtering, and 2) A harmonic analysis of time series (HANTS). These two filtering methods^29^ are applied to a sample data to demonstrate their effectiveness. The data covers the period from 22/03/2018 to 25/06/2018, with time interval of 30 minutes (see Fig. 3).

### Quantile filtering

In quantile filtering, we assume that any data point outside of a defined quantile is an outlier. The filtering procedure is as follows:

1 $$\begin{array}{c}If\,{T}_{b}^{p}(i) > Quantile[{T}_{b}^{p}({\rm{i}}-{\rm{K}}:{\rm{i}}+{\rm{K}}),q]\\ Then\,{loc.append}(i)\,{\rm{\#}}\,record\,position\end{array}$$

2 $$\begin{array}{l}If\,PI(i) > Quantile[PI({\rm{i}}-{\rm{K}}:{\rm{i}}+{\rm{K}}),q]\\ Then\,{loc.append}(i)\,\#\,record\,position\end{array}$$

where ${T}_{b}^{p}$ is the observed brightness temperature with *p* (H, V) polarization. *q* is the quantile (ranging in [0, 1]) to be computed and K is half-time window. In the case of Eq. (1), *q* = 0.85 for ${T}_{b}^{H}$, *q* = 0.90 for ${T}_{b}^{V}$ and K takes the value of 100. For Eq. (2), *PI* is the polarization index calculated as $(({T}_{b}^{V}-{T}_{b}^{H})/({T}_{b}^{V}+{T}_{b}^{H}))$, for which *q* = 0.90 and K takes the value of 100.

### Filtering using the HANTS algorithm

In applying the HANTS^26^ algorithm, we define an outlier as

3 $${T}_{b}^{p}(i) > Maxium[{T}_{b}^{p}{\rm{\_}}HANTS(i-K:i+K),q]$$

where ${T}_{b}^{p}{\rm{\_}}HANTS$ is the estimated brightness temperature by using the HANTS algorithm. The frequency (nf) as one of inputs to the HANTS algorithm is set at 50 in this case. Descriptions of other inputs such as the valid minimum and maximum values can be found in the script (the uploaded HANTS.py)^28^. *q* and K have the same meanings as in Eq. (1), *q* = 0.90 and K takes the value of 150 in this case.

### Filtering results

Supplementary Figure S10 shows results by quantile filtering of ${T}_{b}^{p}$ and Supplementary Figure S11 shows those by quantile filtering of *PI*. Supplementary Figure S12 shows seasonal variations of the Maqu ELBARA-III radiometry dataset for pre-monsoon period, in which the displayed ${T}_{b}^{p}$ are based on quantile filtering of ${T}_{b}^{p}$. Supplementary Figure S13 shows results by using the HANTS algorithm and Supplementary Figure S14 shows seasonal variations of the Maqu ELBARA-III radiometry dataset for the same period, in which the displayed ${T}_{b}^{p}$ in the figure are based on filtering using the HANTS algorithm.

The results in Supplementary Figure S10 appear adequate for outliers with big deviations, however the data points represented by black (blue) dots from 26/03/2018 to 09/04/2018 may contain surface freeze/thaw information, and one may want to keep them from being masked out. Supplementary Figure S11 shows extra ‘outliers’ that have minimum values are also identified while the identified ‘outliers’ that have maximum values are the same as in Supplementary Figure S10. Therefore, Eq. (1) is sufficient for masking outliers caused by solar reflection. Supplementary Figure S14 shows that ${T}_{b}^{p}$ outliers can be effectively detected by using the HANTS algorithm, and the results are better than those shown in Supplementary Figure S12 by quantile filtering of ${T}_{b}^{p}$ (see also Supplementary Figures S11 and S13 for the identified ‘outliers’).

### Supplementary information

Supplementary Figures

## Data Availability

The codes for processing the collected data, for plotting the figures, and for downloading SMAP data are included in the dataset^28^. The codes for filtering the reflected solar signals and for plotting the results are included in the update^29^ together with the updated brightness temperature that contain corrected local time stamps. Overviews of the codes, data and explanations can be found in Online-only Table 2^28^ and Table 2^29^.

## Online-only Tables

**Online-only Table 1:** General characteristics of the Maqu site and included variables in the different data.

Name	Instruments	Variables	Notes
Geographic location		N33.90°, E102.15°, 3432 m a.m.s.l	latitude, longitude, altitude (meter above mean sea level)
Land cover		Grassland	
Climate		Cold climate with dry winter and warm summer (Dwb)	According to updated Köppen-Geiger climate classification^19^
L-band brightness temperature	ELBARA III microwave radiometer (1.4 GHz, Gamma Remote Sensing AG)	Brightness temperature in horizontal and vertical polarization (K)	Incidence angle: 40° to 70^o^ in steps of 5^o^
Profile soil moisture and soil temperature	(SMST_Z) 5TM ECH2O probes (METER Group, Inc. USA)	Soil moisture (m^3^/m^3^) Soil temperature (^o^C)	15-min time intervals, installed at depths: 5, 10, 20, 40, 80 and 160 cm^20^
	(SMST_LC) 5TM ECH2O probes (Decagon Devices, Inc., USA)	Soil moisture (m^3^/m^3^) Soil temperature (^o^C)	Installation depths: 2 sensors at 2.5 cm, then one sensor at every 2.5 cm in the top 20 cm, every 5 cm between 20–50 cm and every 10 cm between 50–100 cm (19 layers and 20 sensors in total)^23^
Turbulent heat fluxes	Integrated Eddy Covariance system (EC150 analyzer, CSAT3A anemometer, HMP155A relative humidity and temperature, and 109-L air temperature) (Campbell scientific, USA)	3-D wind, CO2/H2O fluxes, air temperature and relative humidity	2.5 m height
Radiation	NR01-L pyranometer and pyrgeometer (Hukseflux, NL)	4 component down and upwelling solar and thermal radiation	1.3 m height
Meteorological data	HMP155A air temperature and relative humidity, 109-L air temperature (Campbell scientific, USA)	air temperature, relative humidity	2 m height
	NR01-L pyranometer and pyrgeometer, (Hukseflux)	Four component radiation	1.6 m height
	Windsonic-2D wind speed (Gill)	Wind speed/direction	2 m height
	Precipitation (Geonor T-200B Series)	Liquid precipitation	1.5 m height
Soil and vegetation data	*In-situ* sampling	Soil texture, hydraulic and thermal properties, fresh and dry above-ground biomass, vegetation height, and leaf area index	Soil properties^20^ and vegetation data^27^

**Online-only Table 2:** Overview of the structure and content of the figshare data record^28^ (including directory, subdirectory and file listing, and content).

Directory / Filename	Subdirectory / Filename	Subdirectory / Filename	Content
ELBARA-III dataset_Update			
	**Code**		
		**Plot_TB_SM_Pre**	
		AWS_TB_SMST_Display.py	Code for plotting AWS, TB and SMST (Fig. 6)
		Time_F.py	Code for rounding datetime to any time interval in seconds
		Time_F.pyc	Python interpreter generated file when Time_F.py is imported.
		**MODIS_LAI**	
		HANTS.py	Code for HANTS algorithm
		LAI_Hants_Process.py	Code for smoothing MODIS LAI with HANTS algorithm
		MODIS_LAI_Extract.py	Code for extracting MODIS LAI
		Time_F.py	Code for rounding datetime to any time interval in seconds
		Time_F.pyc	Python interpreter generated file when Time_F.py is imported.
		**SMAP_TB** SMAP L1C TB reading.m	Code for extracting SMAP L1C data
		**AWS_Time_Check**	
		AWS_TB_30min_timeCheck.py	Code for checking time information of AWS data.
		Time_F.py	Code for rounding datetime to any time interval in seconds
		Time_F.pyc	Python interpreter generated file when Time_F.py is imported.
	**Data**	ELBARA-III dataset-2016–2017ELBARA-III TB.csv	ELBARA TB data, 2016–2017
		ELBARA-III dataset-2016–2017Field_Unit.csv	Definition of field (and unit) involved in each.csv file in 2016–2017 period
		ELBARA-III dataset-2016–2017MeteoData_15min_20160325_201701.csv	Meteorological data, 2016–2017, 15 min interval
		ELBARA-III dataset-2016–2017MeteoData_30min_20160101_0315.csv	Meteorological data, 2016–2017, 30 min interval
		ELBARA-III dataset-2016–2017MODIS LAI_HANTS.csv	LAI at hourly scale interpolated from MODIS LAI after smoothing by HANTS algorithm, 2016–2017
		ELBARA-III dataset-2016–2017SMAP TB.csv	SMAP TB data, 2016–2017
		ELBARA-III dataset-2016–2017SMST_LC.csv	SMST_LC data, 2016–2017
		ELBARA-III dataset-2016–2017SMST_Z.csv	SMST_Z data, 2016–2017
		ELBARA-III dataset-2016–2019MODIS_LAI_8daily.csv	MODIS LAI 8-day data, 2016–2019
		ELBARA-III dataset-2017–2018ELBARA-III TB.csv	ELBARA TB, 2017–2018
		ELBARA-III dataset-2017–2018Field_Unit.csv	Definition of field (and unit) involved in each.csv file in 2017–2018 period
		ELBARA-III dataset-2017–2018LAI_*IN SITU*.csv	*In Situ* LAI data, 2018
		ELBARA-III dataset-2017–2018MeteoData_30min.csv	Meteorological data, 2017–2018, 30 min interval
		ELBARA-III dataset-2017–2018MODIS LAI_HANTS.csv	LAI at hourly scale interpolated from MODIS LAI after smoothing by HANTS algorithm, 2017–2018
		ELBARA-III dataset-2017–2018SMAP TB.csv	SMAP TB data, 2017–2018
		ELBARA-III dataset-2017–2018SMST_LC.csv	SMST_LC data, 2017–2018
		ELBARA-III dataset-2018–2019ELBARA-III TB.csv	ELBARA TB, 2018–2019
		ELBARA-III dataset-2018–2019Field_Unit.csv	Definition of field (and unit) involved in each.csv file in 2018–2019 period
		ELBARA-III dataset-2018–2019MeteoData_30min.csv	Meteorological data, 2018–2019, 30 min interval
		ELBARA-III dataset-2018–2019MODIS LAI_HANTS.csv	LAI at hourly scale interpolated from MODIS LAI after smoothing by HANTS algorithm, 2018–2019
		ELBARA-III dataset-2018–2019SMAP TB.csv	SMAP TB data, 2018–2019
		ELBARA-III dataset-2018–2019SMST_LC.csv	SMST_LC data, 2018–2019
		FluxDataAllField2016–2017.csv	Eddy covariance data, 2016–2017
		FluxDataAllField2017–2018.csv	Eddy covariance data, 2017–2018
		FluxDataAllField2018–2019.csv	Eddy covariance data, 2018–2019
	eddycovariance_rawdata_metafile.pdf		Definition of field (and unit) involved in each Eddy covariance data file
	MODIS_Download_Procedure.pdf		Procedure for downloading MODIS data
	README.txt		README File
	SMAP L1C over Maqu ELBARA site.tif		SMAP L1C coverage over Maqu

## References

[1] Kerr YH (2012). The SMOS soil moisture retrieval algorithm. IEEE Trans. Geosci. Remote Sens..

[2] Entekhabi D (2010). The soil moisture active passive (SMAP) mission. Proc. IEEE..

[3] Colliander A (2017). Validation of SMAP surface soil moisture products with core validation sites. Remote Sens. Environ..

[4] Kurum M (2010). A first-order radiative transfer model for microwave radiometry of forest canopies at L-band. IEEE Trans. Geosci. Remote Sens..

[5] Zhao Q, Lang RH (2012). Fresnel double scattering from tree branches. IEEE Trans. Geos. Remote Sens..

[6] Schwank M, Naderpour R, Mätzler C (2018). “Tau-Omega”-and two-stream emission models used for passive L-band retrievals: Application to close-range measurements over a forest. Remote Sens..

[7] Kerr YH (2016). Overview of SMOS performance in terms of global soil moisture monitoring after six years in operation. Remote Sens. Environ..

[8] Wigneron JP (2017). Modelling the passive microwave signature from land surfaces: A review of recent results and application to the L-band SMOS & SMAP soil moisture retrieval algorithms. Remote Sens. Environ..

[9] Chaubell MJ (2020). Improved SMAP dual-channel algorithm for the retrieval of soil moisture. IEEE Trans. Geosci. Remote Sens..

[10] Su Z (2011). The Tibetan Plateau observatory of plateau scale soil moisture and soil temperature (Tibet-Obs) for quantifying uncertainties in coarse resolution satellite and model products. Hydrol. Earth Syst Sci..

[11] Zheng D (2018). Impact of surface roughness, vegetation opacity and soil permittivity on L-band microwave emission and soil moisture retrieval in the third pole environment. Remote Sens. Environ..

[12] Yu L, Zeng Y, Wen J, Su Z (2018). Liquid‐vapor‐air flow in the frozen soil. J. Geophys. Res. Atmos..

[13] Zheng D (2017). L-band microwave emission of soil freeze–thaw process in the third pole environment. IEEE Trans. Geosci. Remote Sens..

[14] Mwangi S, Zeng Y, Montzka C, Yu L, Su Z (2020). Assimilation of cosmic‐ray neutron counts for the estimation of soil ice content on the eastern Tibetan Plateau. J. Geophys. Res. Atmos..

[15] Rautiainen K (2014). Detection of soil freezing from L-band passive microwave observations. Remote Sens. Environ..

[16] Rautiainen K (2016). SMOS prototype algorithm for detecting autumn soil freezing. Remote Sens Environ..

[17] Entekhabi D (2014). SMAP Handbook–Soil Moisture Active Passive: Mapping Soil Moisture And Freeze/Thaw From Space.

[18] Dente L, Vekerdy Z, Wen J, Su Z (2012). Maqu network for validation of satellite-derived soil moisture products. Int. J. Appl. Earth Observ. Geoinform..

[19] Peel MC, Finlayson BL, McMahon TA (2007). Updated world map of the Köppen-Geiger climate classification. Hydrol. Earth Syst. Sci..

[20] Zhao H, Zeng Y, Lv S, Su Z (2018). Analysis of soil hydraulic and thermal properties for land surface modeling over the Tibetan Plateau. Earth Syst. Sci. Data..

[21] Schwank M (2010). ELBARA II, an L-band radiometer system for soil moisture research. Sensors..

[22] Schwank M, Matzler C, Guglielmetti M, Fluhler H (2008). L-band radiometer measurements of soil water under growing clover grass. IEEE Trans. Geosci. Remote Sens..

[23] Lv S, Zeng Y, Wen J, Zhao H, Su Z (2018). Estimation of penetration depth from soil effective temperature in microwave radiometry. Remote Sens..

[24] Topp GC, Davis JL, Annan AP (1980). Electromagnetic determination of soil water content: measurements in coaxial transmission lines. Water Resour. Res..

[25] Myneni, R., Knyazikhin, Y. & Park, T. *MCD15A2H MODIS/Terra+ Aqua Leaf Area Index/FPAR 8-day L4 Global 500m SIN Grid V006*. NASA EOSDIS Land Processes DAAC (2015).

[26] Verhoef W, Menenti M, Azzali S (1996). Cover a colour composite of NOAA-AVHRR-NDVI based on time series analysis (1981-1992). Int. J. Remote Sens..

[27] Hofste, J. G. *et al*. Year-long, broad-band, microwave backscatter observations of an alpine meadow over the Tibetan Plateau with a ground-based scatterometer. *Earth Syst. Sci. Data Discuss*., 10.5194/essd-2020-44 (in review, 2020).

[28] Su Z (2020). figshare.

[29] Su Z (2020). figshare.

[30] Lv S, Zeng Y, Su Z, Wen J (2019). A closed-form expression of soil temperature sensing depth at L-band. IEEE Trans. Geosci. Remote Sens..

[31] Zeng Y (2016). Blending satellite observed, model simulated, and *in situ* measured soil moisture over Tibetan Plateau. Remote Sens..

[32] Zhuang R, Zeng Y, Manfreda S, Su Z (2020). Quantifying long-term land surface and root zone soil moisture over Tibetan Plateau. Remote Sens..

