# Supplementary material for: Multiyear in-situ L-band microwave radiometry of land surface processes on the Tibetan Plateau
Source: Sci Data. 2020 Sep 30;7:317. doi: 10.1038/s41597-020-00657-1 (PMC7527448; doi:10.1038/s41597-020-00657-1)
Supplement: Supplementary file 1 — Supplementary Figures [file 41597_2020_657_MOESM1_ESM.pdf]

Supplementary figures

(Supplementary Information to Su et al., Sci. Data, 2020)

Table of contents

Figure S1. Diurnal dynamics of the Maqu ELBARA-III radiometry dataset for 11/10/2016 – 29/10/2016 post-monsoon period. Plotted variables are the same as in Figure 3, except diurnal characteristics in different seasons are highlighted.

Figure S2. Same as Figure 8 but for 08/08/2017 – 26/08/2017 monsoon period.

Figure S3. Same as Figure 8 but for 24/10/2017 – 14/11/2017 post-monsoon period with snow and freezing events.

Figure S4. Same as Figure 8 but for 14/11/2017 – 08/12/2017 post-monsoon to winter period.

Figure S5. Same as Figure 8 but for 07/12/2017 – 31/12/2017 winter period.

Figure S6. Same as Figure 8 but for 04/03/2018 – 01/04/2018 winter to pre-monsoon period.

Figure S7. Same as Figure 8 but for 02/04/2018 – 30/04/2018 pre-monsoon period.

Figure S8. Same as Figure 8 but for 06/07/2018 – 27/07/2018 monsoon period.

Figure S9. Same as Figure 8 but for 28/07/2018 – 11/08/2018 monsoon period.

Figure S10. Quantile filter of T_B^p. Top panel shows original T_B^p with calculated quantile. Bottom panel shows original T_B^p and identified outliers using information from the top panel.

Figure S11. Top panel shows original T_B^p with calculated quantile. Middle panel shows PI with its quantile. Bottom panel shows original T_B^p and identified outliers using information from the top and middle panels.

Figure S12. Seasonal variations of the Maqu ELBARA-III radiometry dataset for pre-monsoon period, in which the displayed T_B^p are based on quantile filtering in terms of Figure S1. Note Figure S3 corresponds to original data in Figure 3 of Su et al. (2020), Sci. Data.

Figure S13. Original T_B^p and filtered ones using the HANTS algorithm.

Figure S14. Seasonal variations of the Maqu ELBARA-III radiometry dataset for pre-monsoon period (late March to late June), in which the displayed T_B^p are after filtering out 'outliers' using the HANTS algorithm.

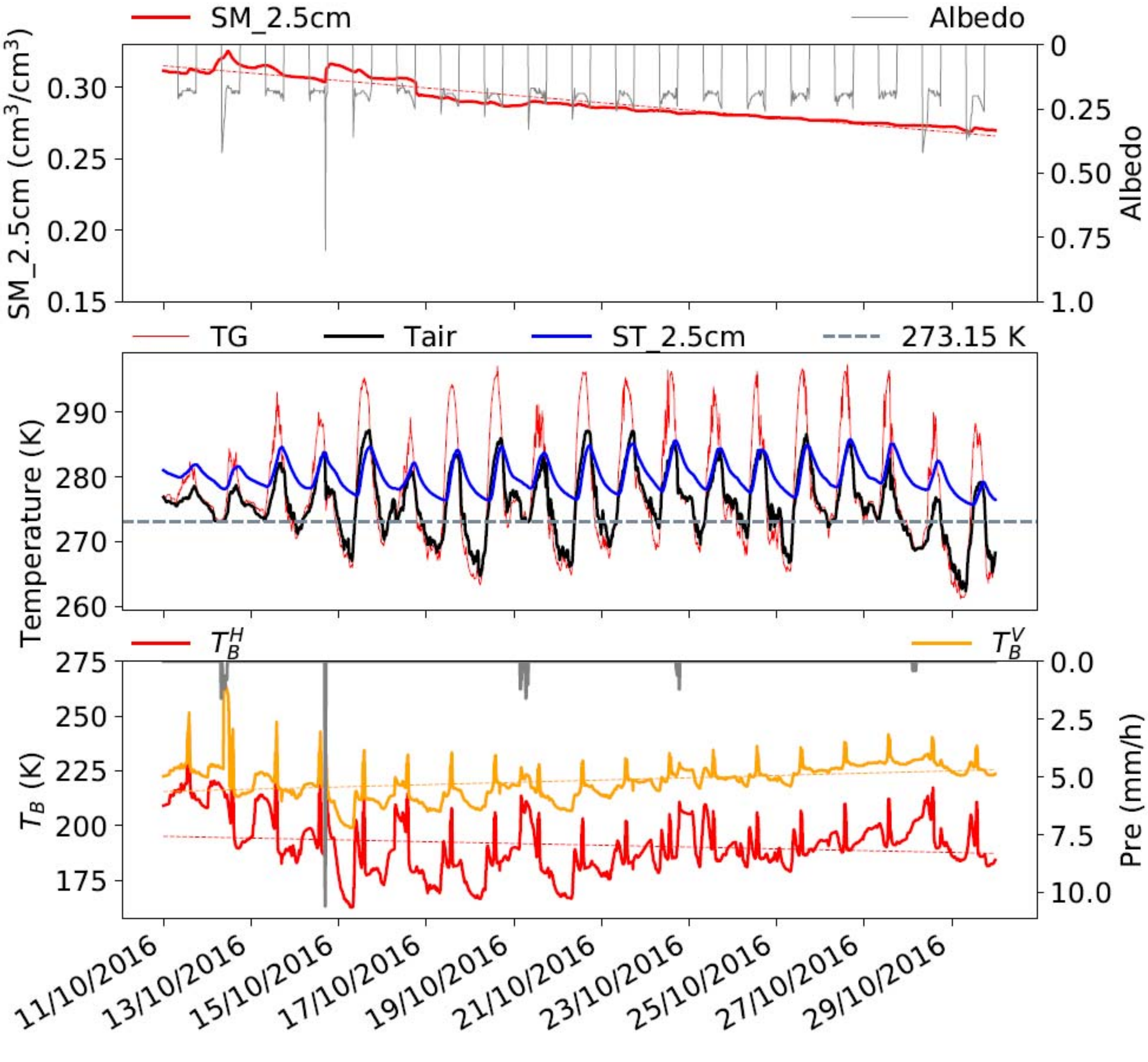

Figure S1. Diurnal dynamics of the Maqu ELBARA-III radiometry dataset for 11/10/2016 – 29/10/2016 post-monsoon period. Plotted variables are the same as in Figure 3, except diurnal characteristics in different seasons are highlighted.

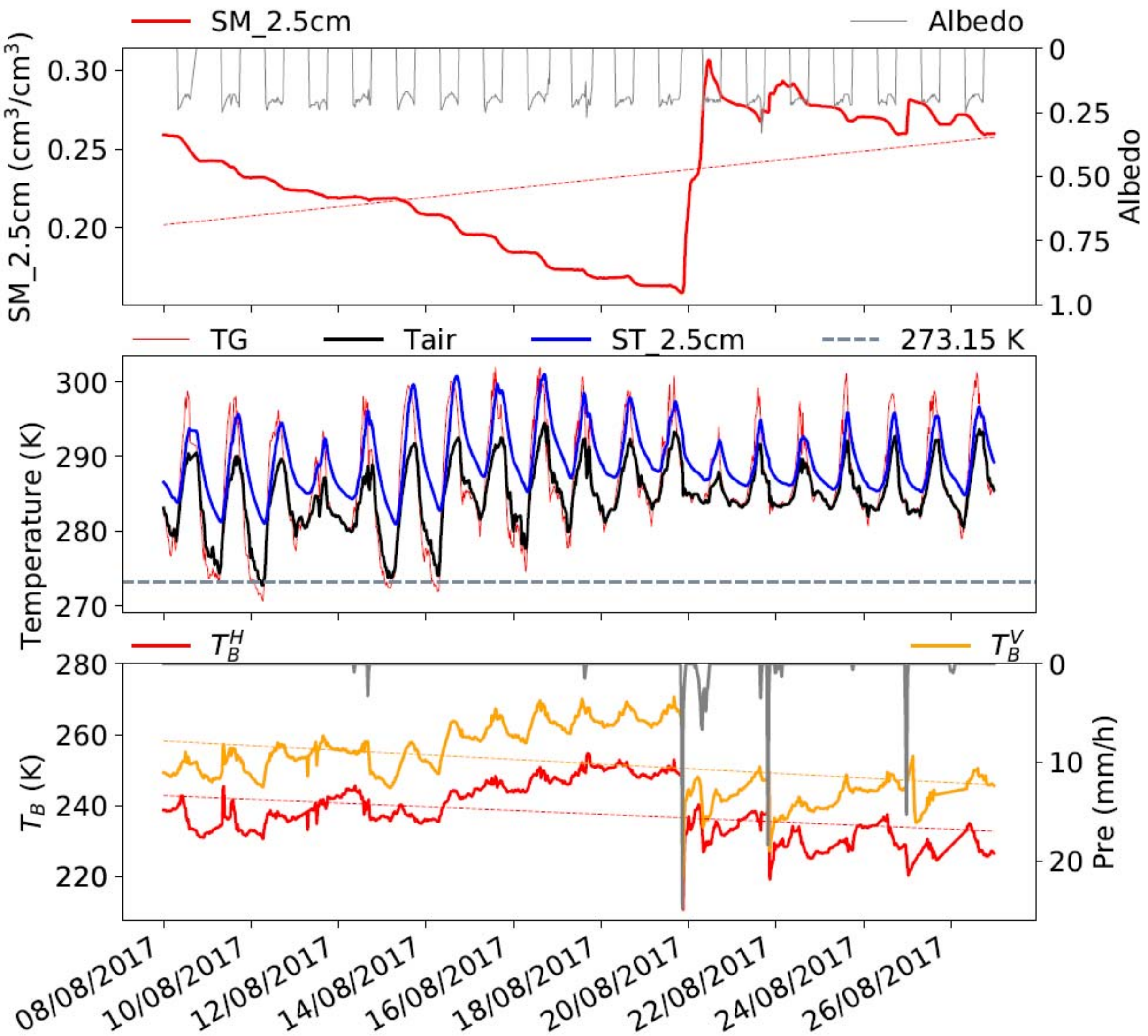

Figure S2. Same as Figure 8 but for 08/08/2017 – 26/08/2017 monsoon period.

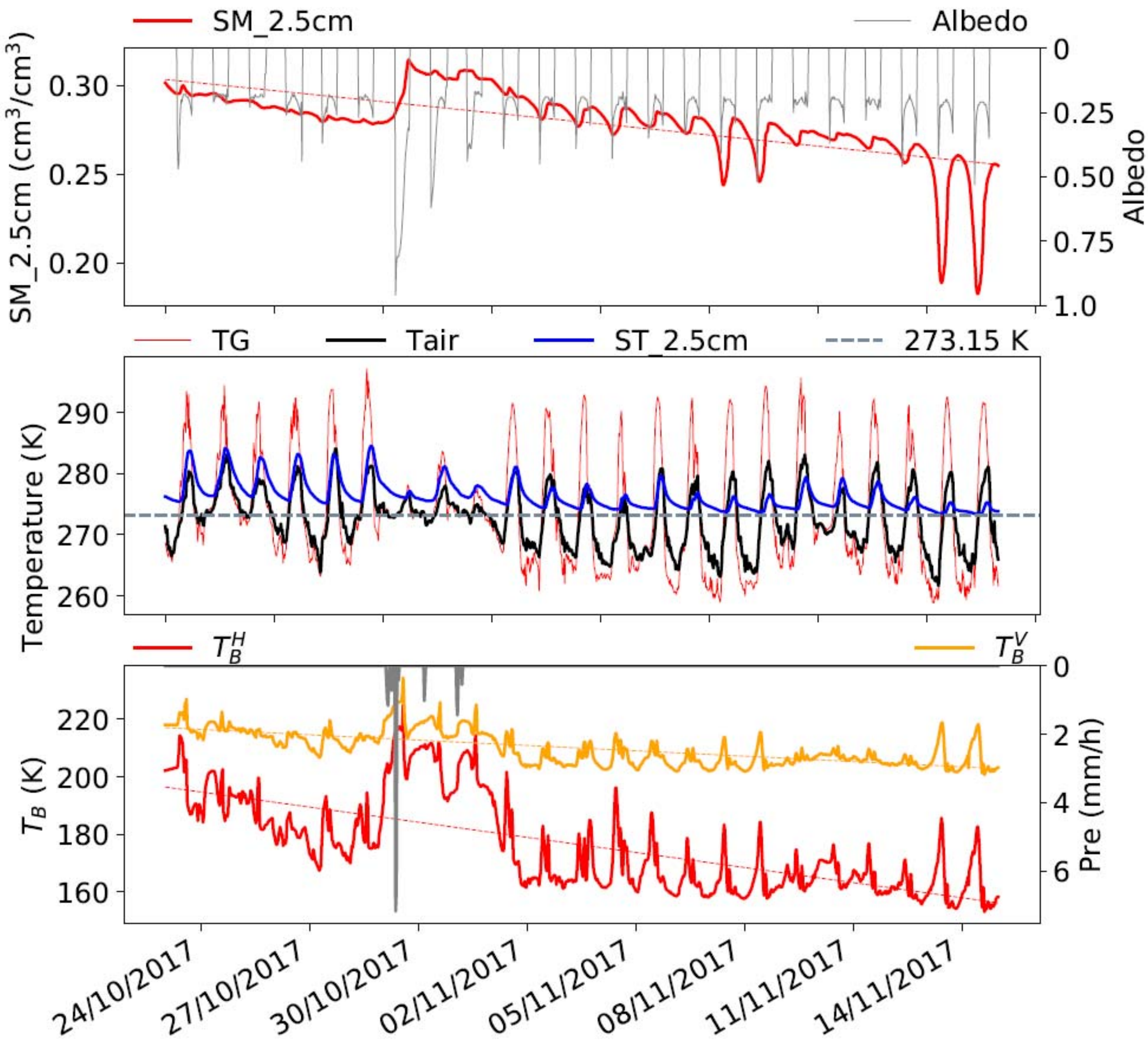

Figure S3. Same as Figure 8 but for 24/10/2017 – 14/11/2017 post-monsoon period with snow and freezing events.

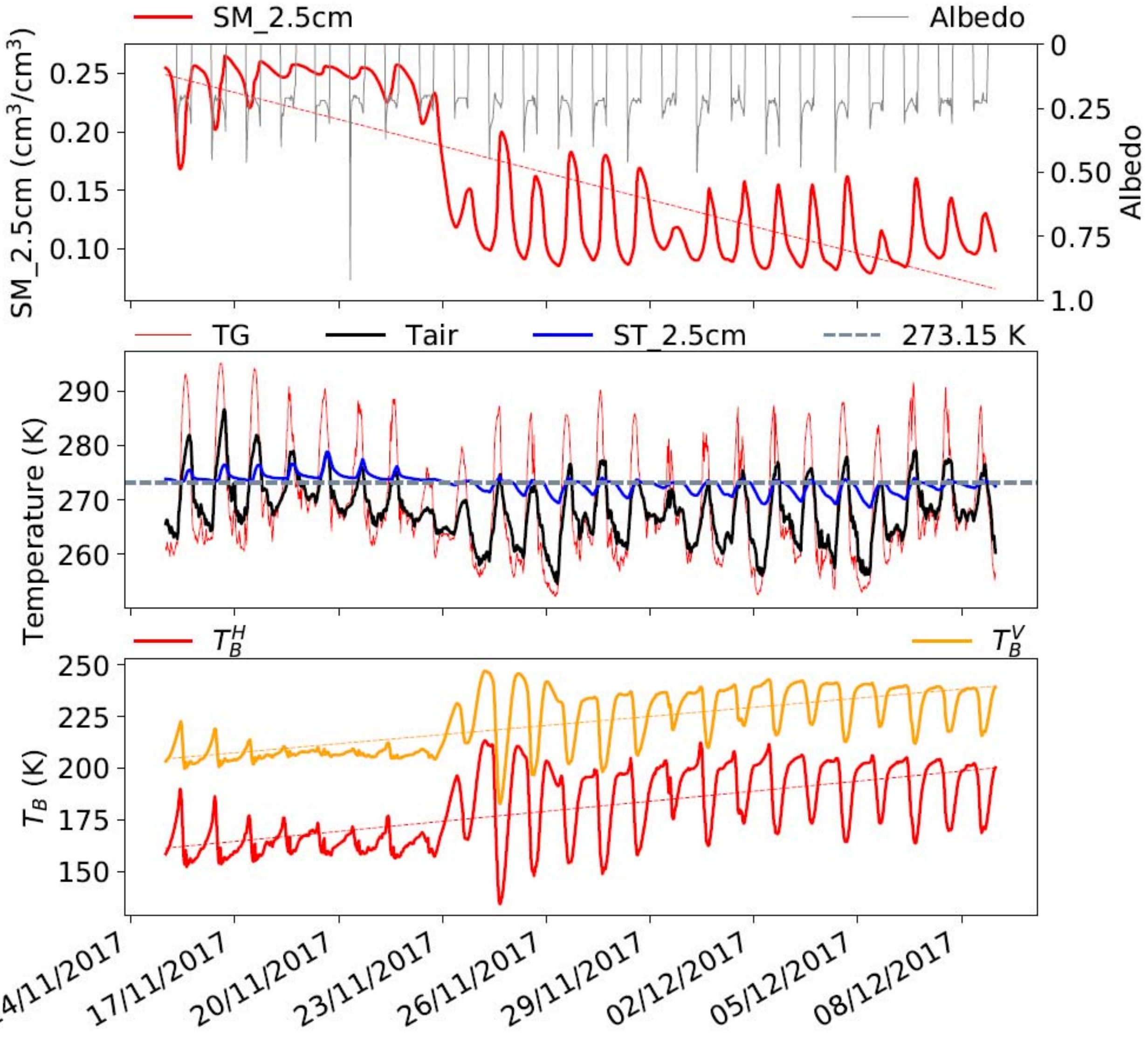

Figure S4. Same as Figure 8 but for 14/11/2017 – 08/12/2017 post-monsoon to winter period.

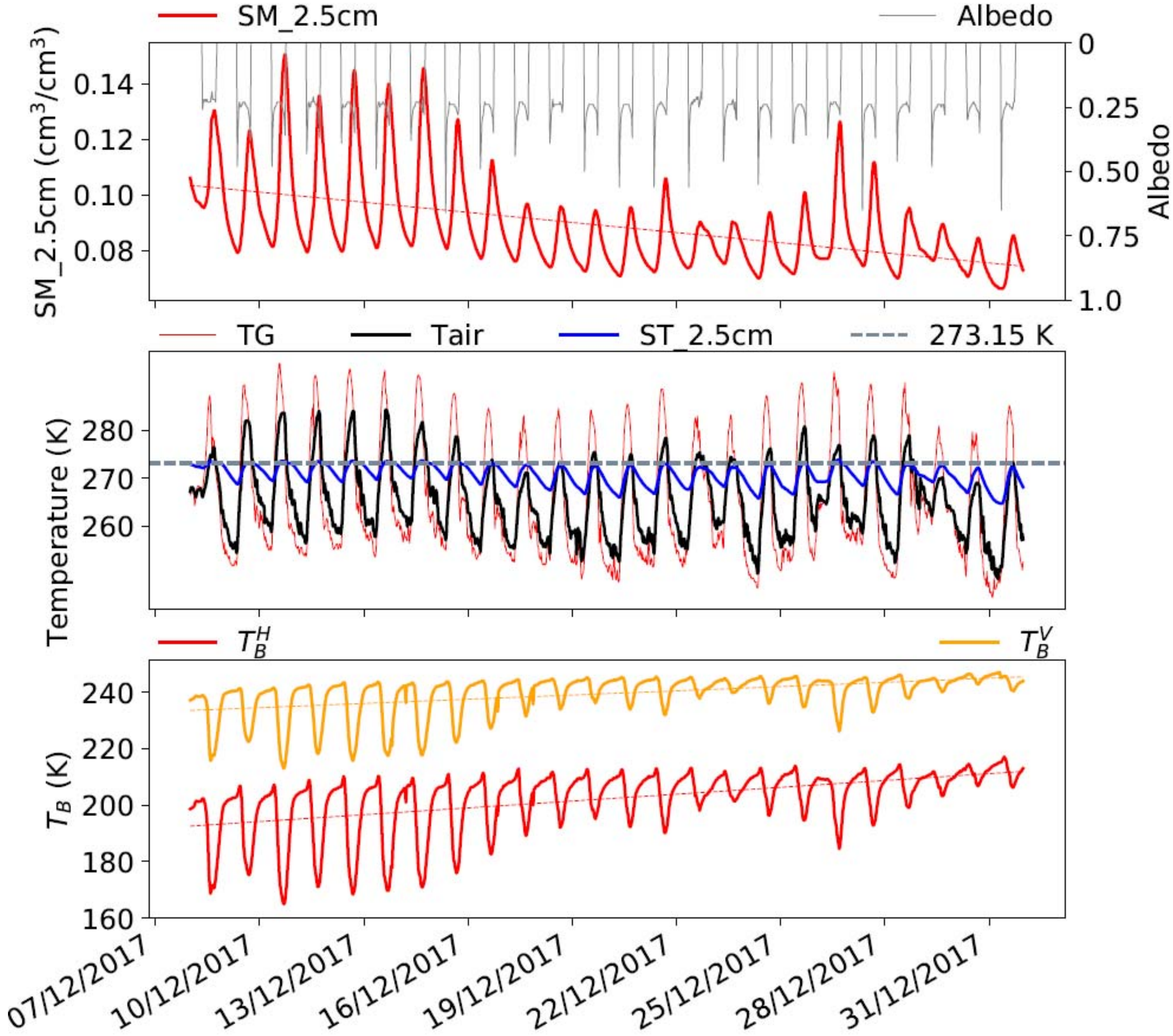

Figure S5. Same as Figure 8 but for 07/12/2017 – 31/12/2017 winter period.

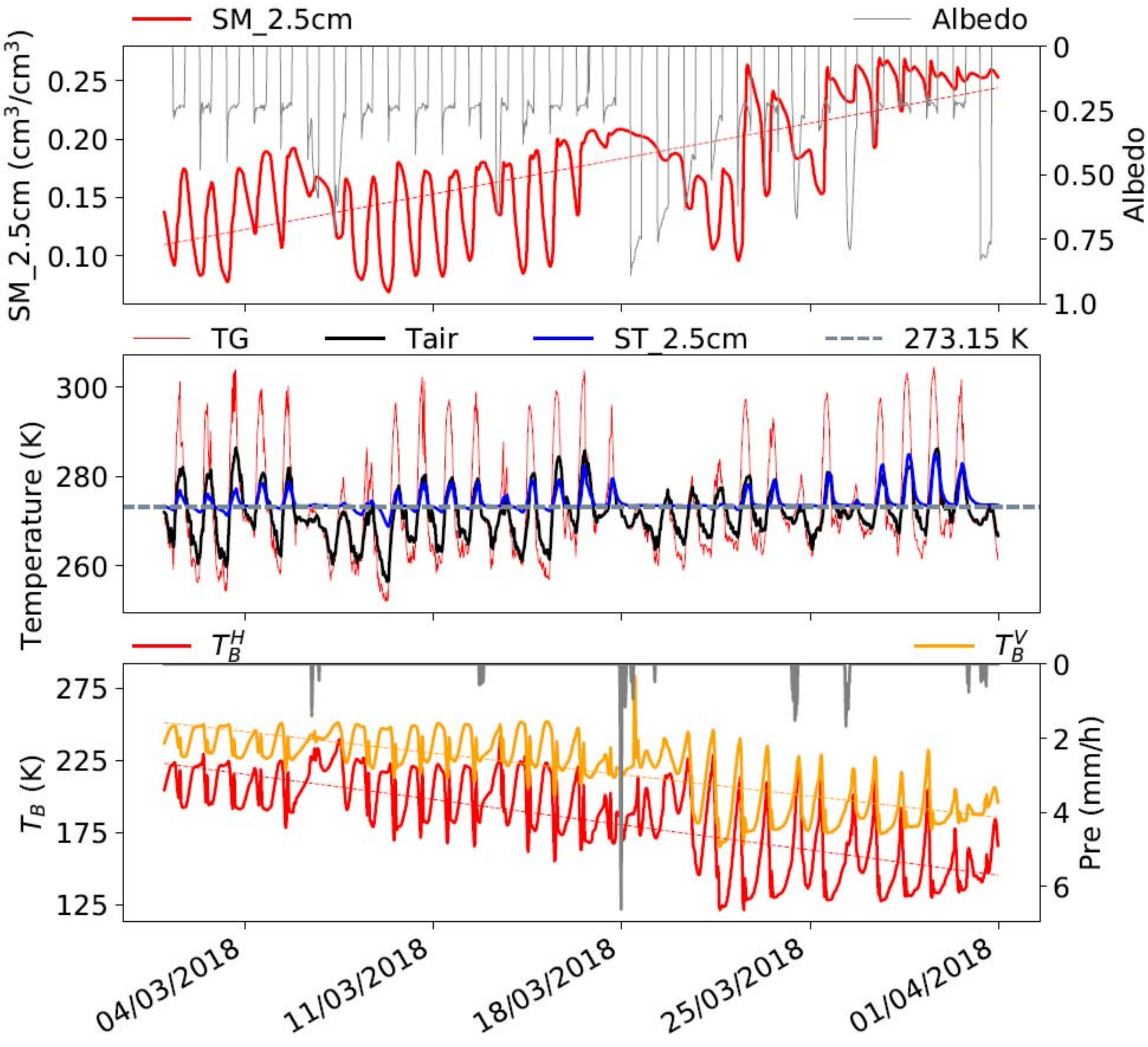

Figure S6. Same as Figure 8 but for 04/03/2018 – 01/04/2018 winter to pre-monsoon period.

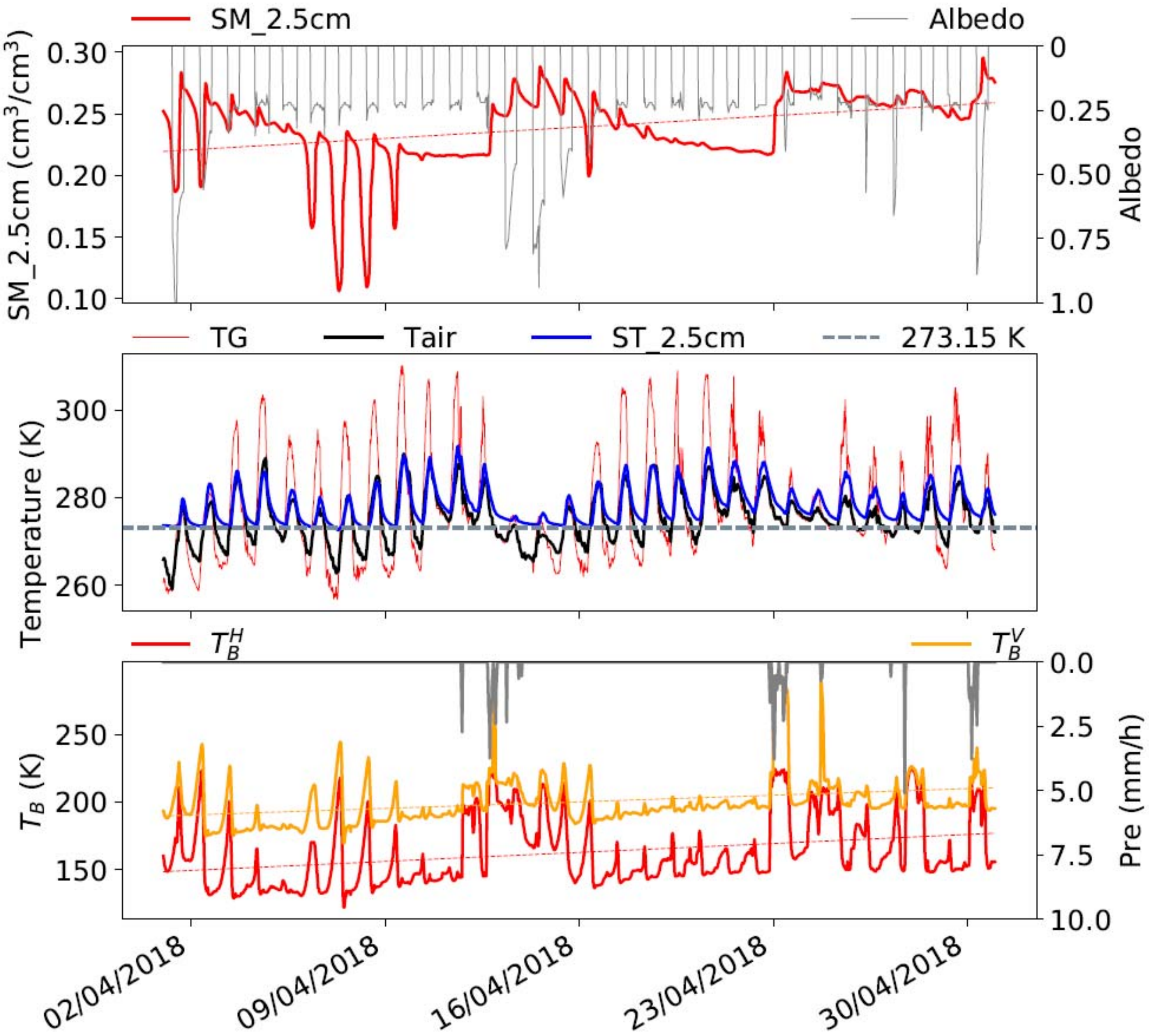

Figure S7. Same as Figure 8 but for 02/04/2018 – 30/04/2018 pre-monsoon period.

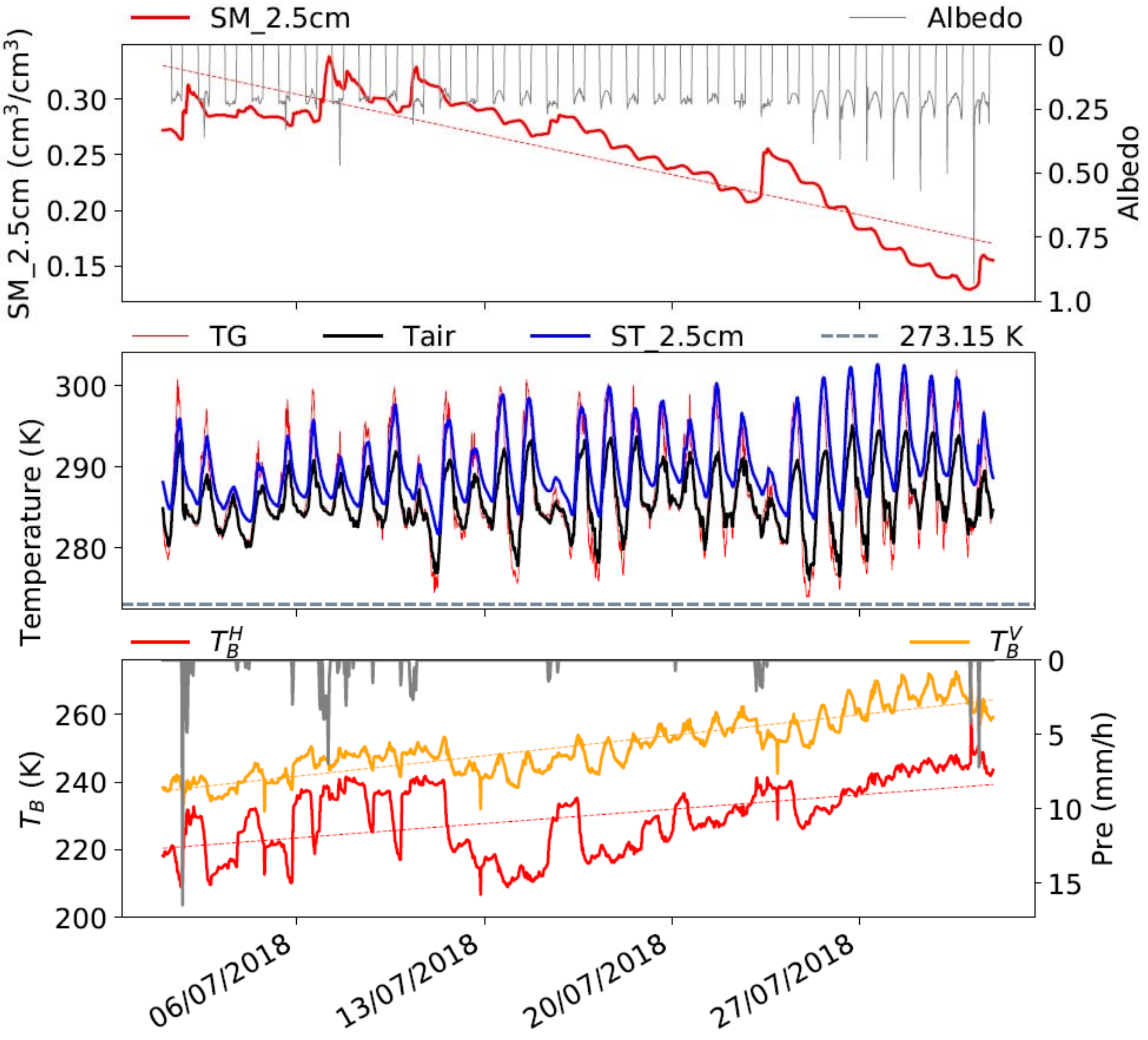

Figure S8. Same as Figure 8 but for 06/07/2018 – 27/07/2018 monsoon period.

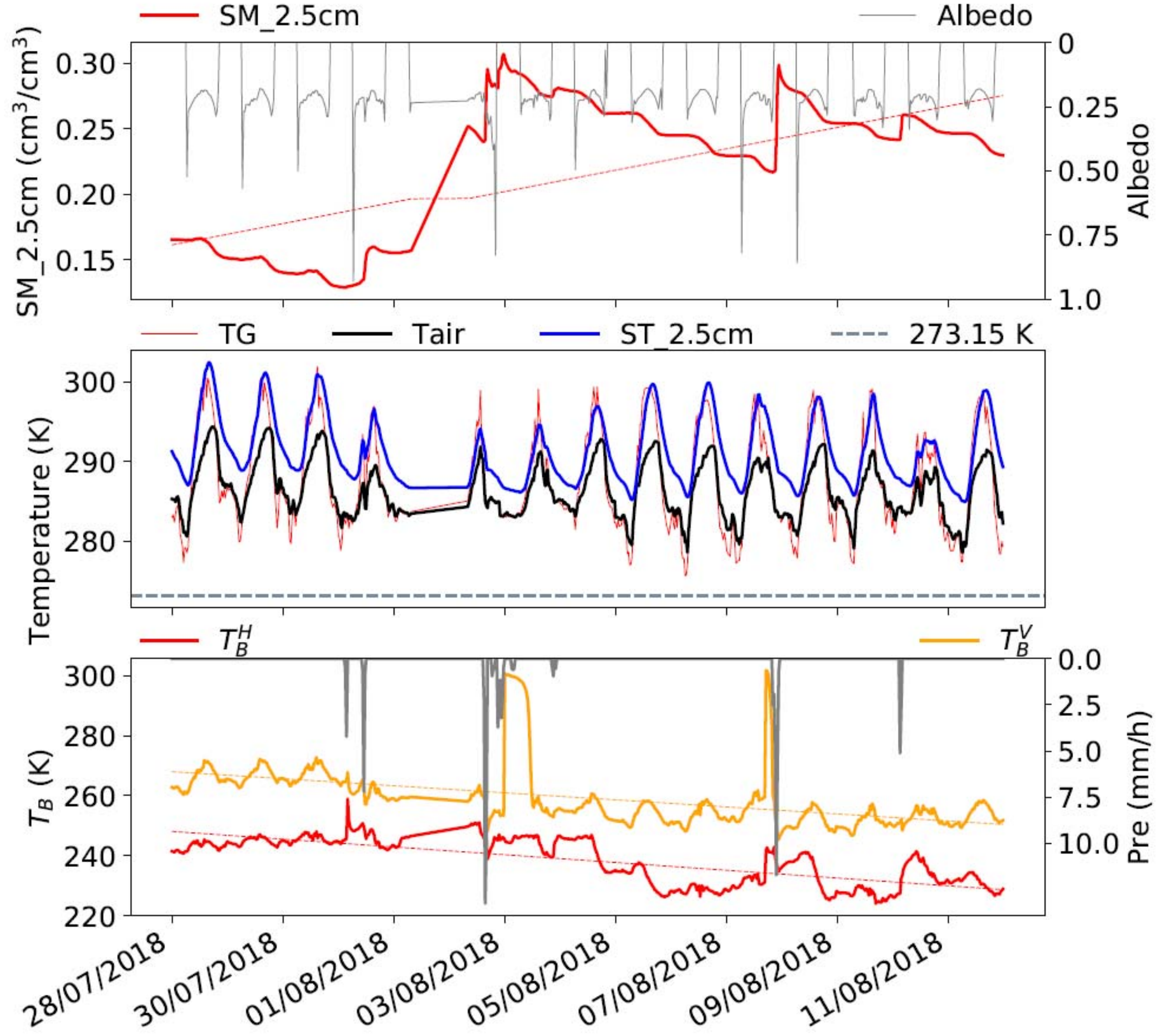

Figure S9. Same as Figure 8 but for 28/07/2018 – 11/08/2018 monsoon period.

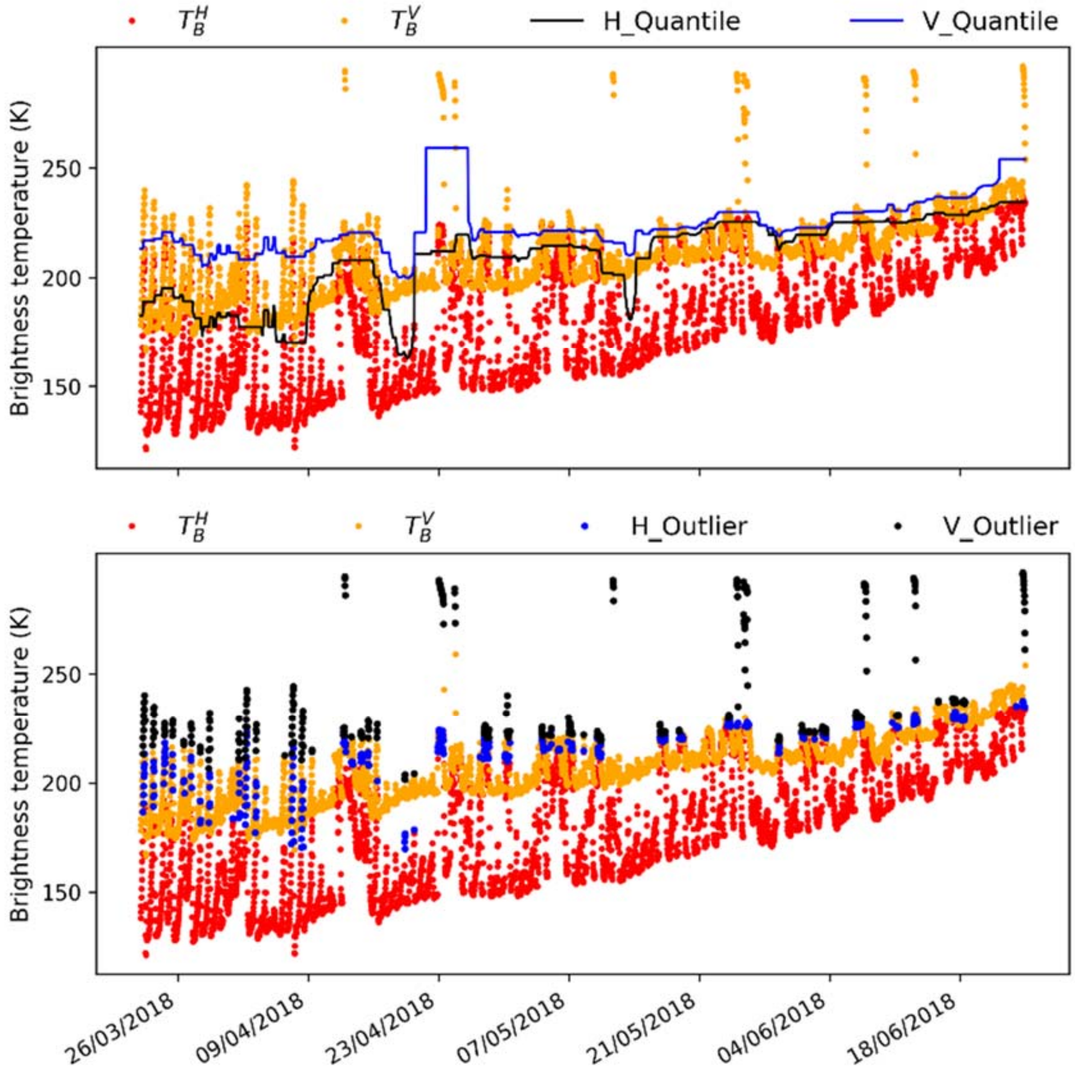

Figure S10. Top panel shows original T_B^p with calculated quantile. Bottom panel shows original T_B^p and identified outliers using information from the top panel.

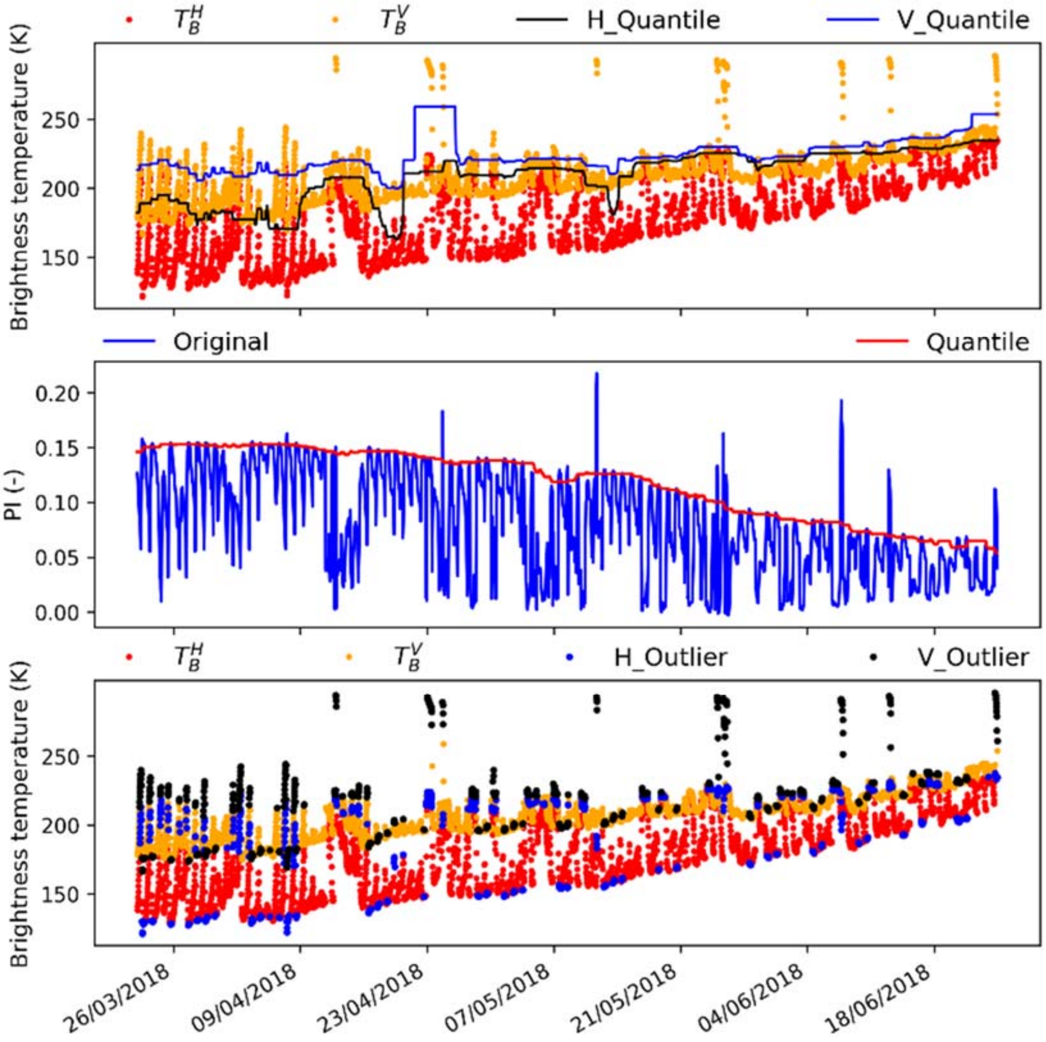

Figure S11. Top panel shows original T_B^p with calculated quantile. Middle panel shows PI with its quantile. Bottom panel shows original T_B^p and identified outliers using information from the top and middle panels.

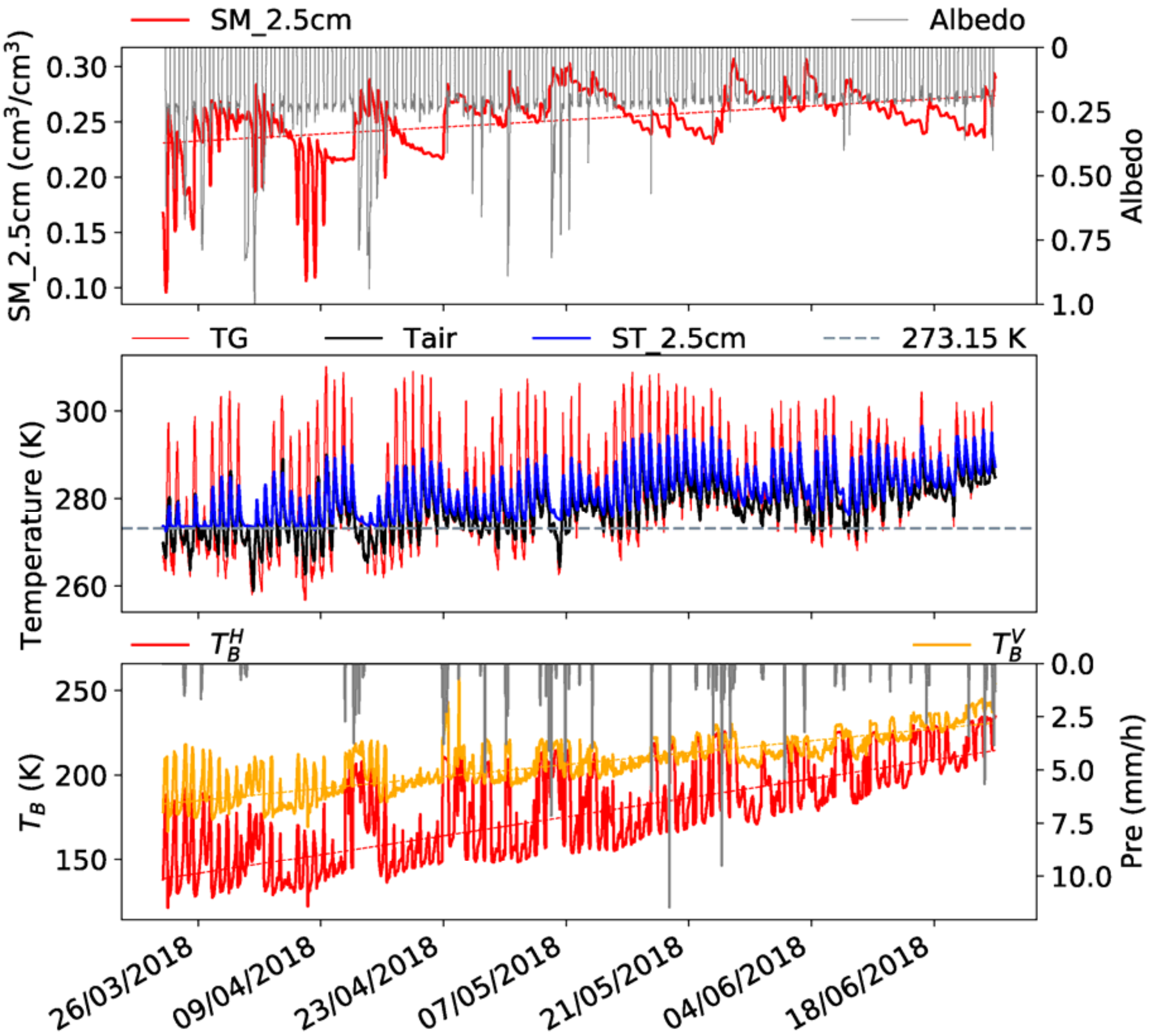

Figure S12. Seasonal variations of the Maqu ELBARA-III radiometry dataset for pre-monsoon period, in which the displayed T_B^p are based on quantile filtering in terms of Figure S1. Note Figure S3 corresponds to original data in Figure 3 of Su et al. (2020), Sci. Data.

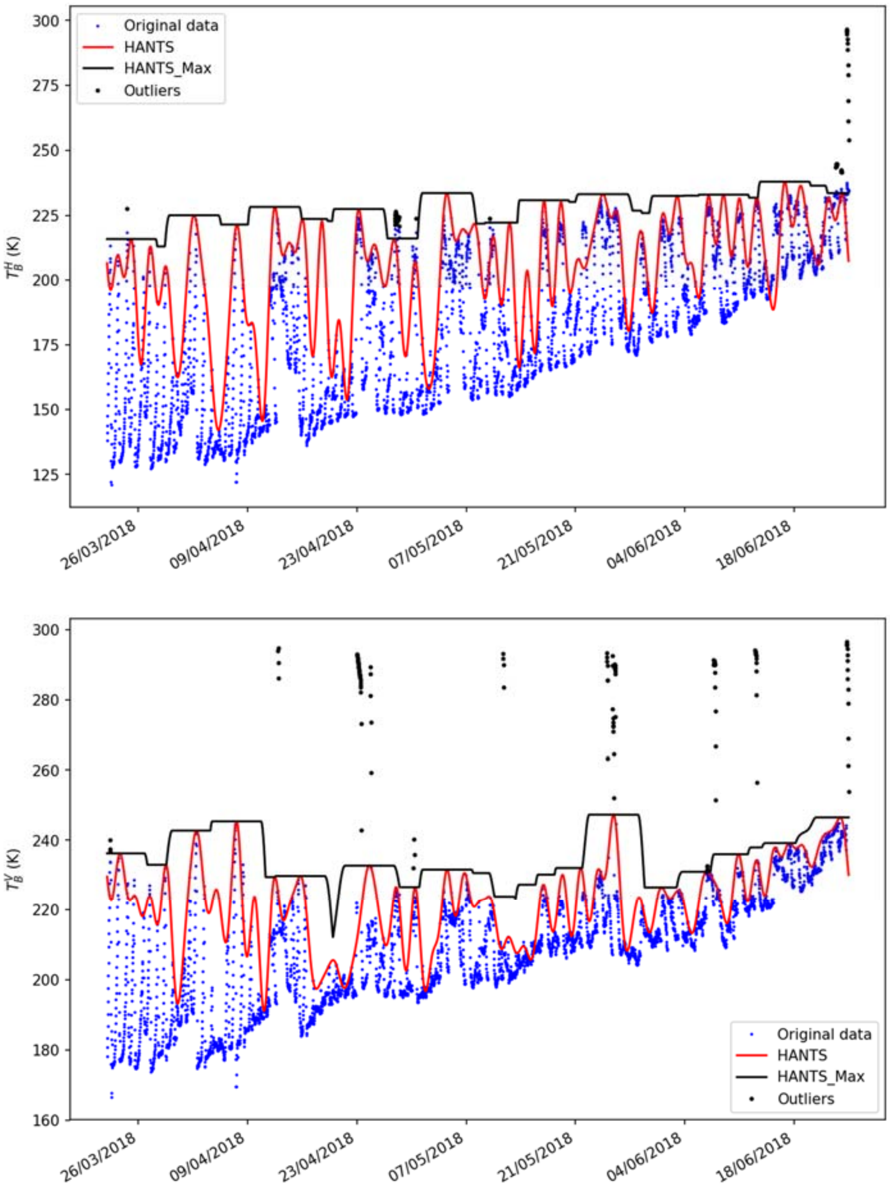

Figure S13. Original T_B^p and filtered ones using the HANTS algorithm.

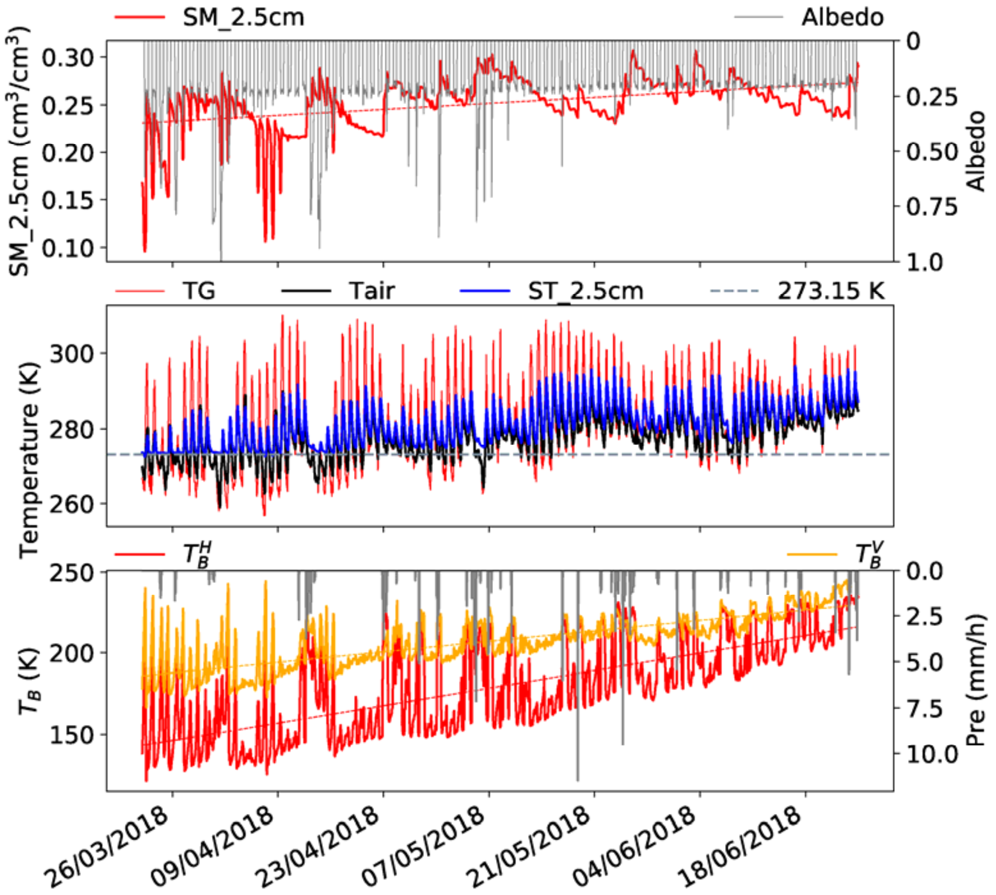

Figure S14. Seasonal variations of the Maqu ELBARA-III radiometry dataset for pre-monsoon period (late March to late June), in which the displayed T_B^p are after filtering out 'outliers' using the HANTS algorithm.
